# Serum clinical laboratory tests and risk of incident dementia: a prospective cohort study of 407,190 individuals

**DOI:** 10.1038/s41398-022-02082-x

**Published:** 2022-08-04

**Authors:** Xiao-Yu He, Kevin Kuo, Liu Yang, Ya-Ru Zhang, Bang-Sheng Wu, Shi-Dong Chen, Wei Cheng, Jian-Feng Feng, Jin-Tai Yu

**Affiliations:** 1 Department of Neurology and National Center for Neurological Disorders, Huashan Hospital, State Key Laboratory of Medical Neurobiology and MOE Frontiers Center for Brain Science, Shanghai Medical College, Fudan University, Shanghai, China; 2 The Institute of Science and Technology for Brain-inspired Intelligence, Fudan University, Shanghai, China

**Keywords:** Predictive markers, Molecular neuroscience

## Abstract

Prevention of dementia is a public health priority, and the identification of potential biomarkers may provide benefits for early detection and prevention. This study investigates the association of common serum laboratory tests with the risk of incident dementia. Among 407,190 participants from the UK Biobank (median follow-up of 9.19 years), we investigated the linear and nonlinear effects of 30 laboratory measures on the risk of all-cause dementia using Cox models and restricted cubic spline models. We found that dementia incidence was associated with low vitamin D concentration (hazard ratio 0.994, 95% confidence interval 0.993–0.996), indicators of endocrine disorders: IGF-1 level (*P* for non-linearity = 1.1E-05), testosterone level (*P* for non-linearity = 0.006); high sex-hormone-binding globulin level (HR 1.004, 95% CI: 1.003–1.006); reduced liver function: lower alanine aminotransferase (HR 0.990, 95% CI: 0.986–0.995); renal dysfunction: cystatin C level (*P* for non-linearity = 0.028); oxidative stress: lower urate level (HR 0.998, 95% CI: 0.998–0.999); lipids dysregulation: lower LDL (HR 0.918, 95% CI: 0.872–0.965) and triglycerides (HR 0.924, 95% CI: 0.882–0.967) concentrations; insulin resistance: high glucose (HR 1.093, 95% CI: 1.045–1.143) and HbA1c (HR 1.017, 95% CI: 1.009–1.025) levels; immune dysbiosis: C−reactive protein (*P* for non-linearity = 5.5E-09). In conclusion, markers of vitamin D deficiency, GH-IGF-1 axis disorders, bioactive sex hormone deficiency, reduced liver function, renal abnormalities, oxidation, insulin resistance, immune dysbiosis, and lipids dysregulation were associated with incident dementia. Our results support a contributory role of systemic disorders and diverse biological processes to onset of dementia.

## Introduction

Dementia is a collection of largely irreversible neurological illnesses characterized by memory loss, cognitive impairment, and difficulties in activities of daily living, which significantly reduce the quality of life and pose a substantial social burden [1]. With the increasing prevalence of dementia and lack of effective treatments [2], current trends show that identifying novel biomarkers and searching for modifiable risk factors to counteract the dementia epidemic with non-invasive methods are in demand [3]. So far, the most frequently mentioned in published studies are inflammatory markers such as interleukin 6 (IL-6), cytokines and C-reactive protein (CRP) [4, 5], neurotrophic factor, immunometabolic markers [6, 7], liver enzymes [8, 9], and metabolism damage markers [10], some of which may reflect comorbid diseases but are closer to the potential pathophysiology and less susceptible to diagnostic bias. Nevertheless, establishing robust and reliable indicators remains a challenge, possibly due to publication bias, confounding, small sample size, insufficient follow-up time, and neglect of possible nonlinear relationship. As dementia is a systemic disease associated with dysfunction in oxidative, inflammatory, and biochemical pathways in peripheral tissues [10], research including full-scale serum laboratory tests may provide evidence for exploring the pathophysiology and identifying the biomarkers of dementia. Additionally, the effects of common laboratory tests have been examined in fields other than dementia [11].

The present work utilizes UK Biobank (UKB) data to comprehensively explore which systemic disorders that might predispose to dementia onset, providing robust findings that overcome traditional limitations. We aim to investigate the associations of biomarkers from common serum laboratory tests and incident dementia. We also evaluate the linear and nonlinear relationships between clinical serum laboratory tests and incident dementia to unveil the previously undetected biomarkers or biological pathways.

## Methods

### Data source and participants

**Fig. 1:**
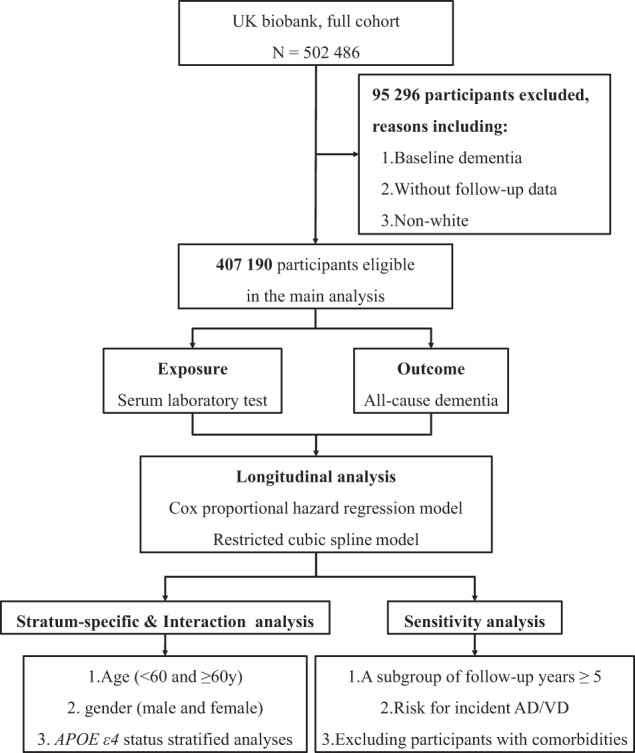
Flow chart of the study design. Flow chart depicting the design and analytic approach of this study in detail.

Participants of this study were a part of the UK Biobank (www.ukbiobank.ac.uk), a large prospective cohort study consisting of 502,617 British males and females (aged 40–69 years at baseline) from 22 assessment centers between 2006 and 2010 [12]. UKB received ethical approval from the National Information Governance Board for Health and Social Care and the National Health Service North West Multi-Center Research Ethics Committee [13]. All participants gave informed consent through electronic signature before enrollment in the study. Analyses were conducted under UKB application number 1954. 70,647 participants were excluded due to already having a dementia diagnosis at baseline or without follow-up. We included participants of self-reported European ancestry only, yielding 407,190 individuals in the final analysis cohort (Fig. 1).

### Dementia diagnosis

The International Classification of Diseases ICD-9 codes (290, 291.2, 294.1, 331.0–331.2, 331.5, 331.0, and 290.4) and ICD-10 codes (A81.0, F00, F01, F02, F03, F05.1, F10.6, G31.0, G31.1, and G31.8) were used to identify all-cause dementia, which was ascertained using a primary or secondary diagnosis in the health records or an underlying cause of death in the death register. A subset of the population was also retrieved from primary care data using read codes (version 2 [Read v2] and version 3 [CTV3 or Read v3]). The date and source of initial diagnoses were derived from the first occurrence categories of mental and behavioral disorders (Fields 130836–130843) and nervous system disorders (Fields 131036–131037) in the UKB.

### Serum laboratory tests

UKB has embarked on a project to measure 30 key serum biochemical markers in biological samples collected at baseline (2006–2010) in all 500,000 participants (Data-Fields tab of https://biobank.ctsu.ox.ac.uk/crystal/label.cgi?Id=17518). Rigorous quality control (QC) and correction were performed for technical outliers (details available at https://biobank.ctsu.ox.ac.uk/crystal/crystal/docs/serum_biochemistry.pdf).

Each blood biochemistry test was categorized as either “bone and joint” (i.e., alkaline phosphatase, calcium, rheumatoid factor, vitamin D), “cancer” (i.e., sex-hormone-binding globulin, testosterone, IGF-1, oestradiol), “cardiovascular” (i.e., CRP, apolipoproteins A and B, lipoprotein A, triglycerides, total cholesterol, HDL cholesterol, LDL cholesterol), “Diabetes” (i.e., glucose, HbA1c), “liver” (i.e., alanine and aspartate aminotransferase, gamma glutamyltransferase, albumin, direct bilirubin, total bilirubin), or “renal” (i.e., cystatin C, creatinine, phosphate, total protein, urate, urea) following the UK Biobank (https://biobank.ctsu.ox.ac.uk/~bbdatan/biomarkers.pdf). We renamed the “cancer” category to “endocrine” because all the markers in it are also endocrine markers. Then we integrated “cardiovascular” and “diabetes” into a single “immunometabolic” category. Undoubtedly, these categories are somewhat oversimplified because some markers can reflect multiple categories that are too hard to classify.

### Covariates

The following covariates were measured at baseline. Demographic variables included age (Field 21022), sex (Field 31), apolipoprotein E *(APOE) ε4* carrier status (carrier/non-carrier status as defined by genetic information), and location of the UKB assessment center (Field 54). Temporal variables included season and time of day of the blood draw (Field 3166) and fasted hours before the blood draw (Field 74). Socioeconomic variables included education, categorized as higher (college/university degree or other professional qualification) or lower (Field 6138), and townsend deprivation index (Field 189). Lifestyle variables included alcohol intake frequency (Field 1558), smoking status (Field 20116), and body mass index (Field 21001). Medication variables included cholesterol-lowering medication use, and insulin use (Field 6153/6177) [11].

### Statistical analysis

Continuous variables were presented using means (standard deviations, SDs) or median (interquartile range, IQR), and categorical variables were described using number (percentage, *N* (%)) according to incident dementia status after follow-up. The outliers more than four times standard deviation from the mean were excluded to avoid the extreme values’ effects. A flow chart of study design overview is shown in Fig. 1. Cox proportional hazards regression was applied to estimate the associations between each blood test and incident dementia, and we used two models to estimate the associations. Follow-up time was calculated as months from baseline to the date of first diagnosis, death, loss to follow-up, or the final date with accessible information from hospital admission, whichever came first. Model 1 was adjusted for demographic and temporal variables, and model 2 was further adjusted for socioeconomic, lifestyle, and medication variables. Model 2 was chosen as the priority model. Nonbinary covariates were standardized to zero mean and unit variance. Proportional hazards were tested using scaled Schoenfeld’s residuals, although there was no indication of a violation of the consumption (global Schoenfeld test *p* > 0.05).

Restricted cubic spline models fitted for Cox proportional hazards models with four knots were used to flexibly model and visualize the relation of each blood test with incident dementia. The spline models were adjusted for the same covariates as in model 2. Potential non-linearity was tested using a likelihood ratio test comparing the model with only a linear term against the model with linear and cubic spline terms.

In sensitivity analyses, firstly, we performed stratum-specific analyses to estimate the associations of each blood test with incident dementia based on age at baseline (≤60 and >60 years), gender (male and female), and *APOE ε4* carrier status (carrier and non-carrier). We also used additional interaction analyses to examine the potential synergistic effect by modeling the product term of the stratifying variable with each serum laboratory test. Secondly, participants with a follow-up time of more than 5 years were analyzed independently to minimize the possible reverse causation bias. Thirdly, we reanalyzed each serum laboratory test with Alzheimer’s disease (AD) and vascular dementia (VD), two most common types of dementia [1], to further explore whether there are differential associations between different kind of dementia. Additionally, we excluded individuals with pre-existing diseases which can influence the corresponding indicators to minimize bias from unbalanced comorbidities. For indicators in differential category, we excluded individuals with different ICD-10 codes (See detail in supplementary Table 9).

All statistical analyses and figure preparations were performed using R software (version 4.1.0). Bonferroni correction was implemented across the tests conducted (30 blood tests), and statistical significance was set at a *P* < 0.05.

## Results

### Baseline results

At baseline, 407,190 European individuals who were free from dementia at baseline from the UKB were included in the main analyses. Among those, the mean age was 57.20 (standard deviation: 7.95) years; 220,714 participants (54.2%) were women. During a median follow-up of 9.19 years (interquartile range: 7.16–10.78), a total of 5334 incident dementia events were recorded. Baseline characteristics of the participants stratified by incident dementia status are shown in Table 1. Compared to individuals without incident dementia, individuals with incident dementia tended to be older, male, *APOE ε4* carriers, and smokers; were more likely to take cholesterol-lowering medications, and insulin. They also had poor socioeconomic conditions and lower education levels.

**Table 1:** Baseline characteristics of study participants by incident dementia status.

Characteristics	Overall	No incident dementia	Incident dementia	*P* value
N	407190	401856	5334	
Age, mean (SD), *y*	57.20 (7.95)	57.10 (7.94)	64.38 (4.67)	<0.001
Gender, *n* (%)				<0.001
Female	220714 (54.2)	218210 (54.3)	2504 (46.9)	
Male	186476 (45.8)	183646 (45.7)	2830 (53.1)	
Townsend Deprivation Index, mean (SD)	−1.42 (3.01)	−1.42 (3.01)	−1.01 (3.30)	<0.001
BMI, mean (SD), kg/m^2^	27.54 (4.83)	27.54 (4.83)	27.81 (5.01)	<0.001
*APOE* ϵ*4*, *n* (%)				<0.001
Carrier	263783 (71.4)	261580 (71.7)	2203 (46.1)	
Non-carrier	105912 (28.6)	103337 (28.3)	2575 (53.9)	
Education, *n* (%)				<0.001
Low	138773 (42.6)	137181 (42.5)	1592 (49.0)	
High	187264 (57.4)	185609 (57.5)	1655 (51.0)	
Smoking status, *n* (%)				<0.001
Never smoked	216656 (53.2)	214232 (53.3)	2424 (45.4)	
Former smoker	146933 (36.1)	144611 (36.0)	2322 (43.5)	
Current smoker	43601 (10.7)	43013 (10.7)	588 (11.0)	
Cholesterol lowering medications, *n* (%)	74657 (18.3)	72704 (18.1)	1953 (36.6)	<0.001
Insulin, *n* (%)	4897 (1.2)	4680 (1.2)	217 (4.1)	<0.001
Vitamin D, mean (SD), nmol/L	49.34 (20.72)	49.36 (20.72)	47.98 (20.98)	<0.001
IGF-1, mean (SD), nmol/L	21.22 (5.54)	21.24 (5.53)	20.11 (5.79)	<0.001
SHBG, mean (SD), nmol/L	50.92 (25.43)	50.89 (25.43)	52.90 (25.06)	<0.001
Testosterone, mean (SD), nmol/L	6.57 (6.02)	6.56 (6.02)	7.33 (6.00)	<0.001
C-reactive protein, mean (SD), mg/L	2.33 (2.84)	2.33 (2.83)	2.51 (3.10)	<0.001
Glucose, mean (SD), mmol/L	5.03 (0.78)	5.03 (0.78)	5.21 (0.93)	<0.001
Glycated haemoglobin, mean (SD), mmol/mol	35.66 (4.88)	35.64 (4.86)	37.47 (5.96)	<0.001
LDL direct, mean (SD), mmol/L	3.56 (0.87)	3.56 (0.87)	3.41 (0.95)	<0.001
Alanine aminotransferase, mean (SD), U/L	22.94 (11.10)	22.95 (11.11)	21.99 (10.05)	<0.001
Creatinine, mean (SD), umol/L	71.96 (14.53)	71.93 (14.50)	73.85 (16.40)	<0.001
Cystatin C, mean (SD), mg/L	0.91 (0.15)	0.91 (0.14)	0.97 (0.17)	<0.001
Urate, mean (SD), umol/L	310.00 (80.17)	309.90 (80.13)	317.38 (82.49)	<0.001
Urea, mean (SD), mmol/L	5.42 (1.29)	5.42 (1.29)	5.68 (1.49)	<0.001

Values are mean (standard deviation) or numbers (percentage). P-values are derived using either Student’s *t*-test or Chi-square test.

Laboratory tests have significant linear or nonlinear association with dementia in the main analysis were shown here, see all 30 laboratory tests baseline characters in supplementary Table 1.

*BMI* body mass index, *APOE* apolipoprotein.

### Association of serum laboratory tests with incident dementia

**Fig. 2:**
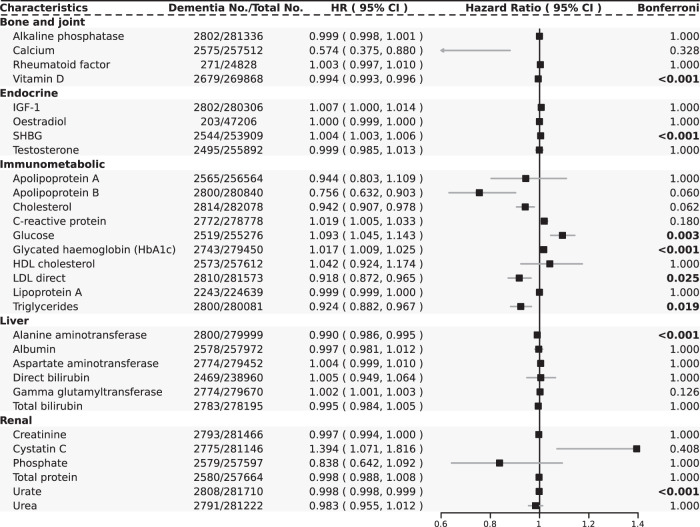
Linear associations between serum laboratory tests and risk of incident dementia. The hazard ratios present here were calculated by the Cox model 2. Bonferroni column in bold indicates statistical significance at a Bonferroni adjusted *p* < 0.05.

In Cox model 2, among the four markers classified as the “bone and joint” category (Fig. 2), lower vitamin D level (HR 0.994, 95% CI: 0.993–0.996, *p* = 1.7E-06) were significantly associated with an increased risk of incident dementia, while alkaline phosphatase, calcium, and rheumatoid factor levels were not. Lower vitamin D level remained strongly associated with incident dementia when restricted the follow-up time to more than 5 years (HR 0.995, 95% CI: 0.993–0.997, *p* = 3.2E-04; Supplemental Table 6). Since low vitamin D levels can represent not only dysregulated skeletal homeostasis but also some specific conditions like kidney disease, or an indicator of poor health, we could not mechanically link bone disorders with dementia.

**Fig. 3:**
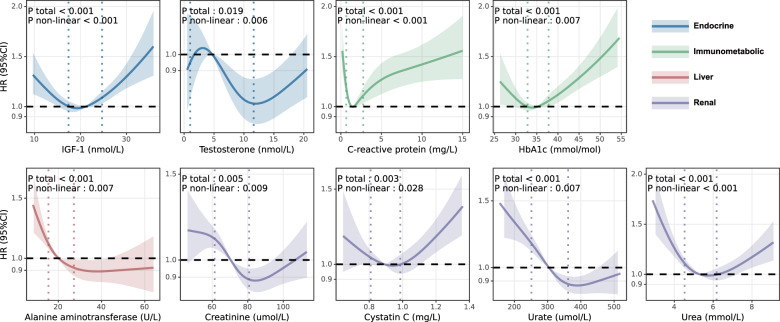
Nonlinear associations between serum laboratory tests and risk of incident dementia. Restricted cubic spline models fitted for Cox proportional hazards models with four knots for 9 significant nonlinear associations from the main analysis (The remaining 21 non-significant associations are shown in Supplementary Fig. 2) Two dashed vertical lines represent 25% and 75% values of each exposure. Results were adjusted for demographic, temporal, socioeconomic, lifestyle and medication variables. The blue, green, red and purple indicates each of serum laboratory tests fitting into “endocrine”, “immunometabolic”, “liver” and “renal” category. CI confidence interval, HR hazard ratio.

Among the four laboratory tests under the “endocrine” category (Fig. 2), there was evidence to show that high sex hormone-binding globulin level (SHBG, HR 1.004, 95% CI: 1.003–1.006, *p* = 8.6E-05) was associated with an increased risk of incident dementia. Beyond that, the restricted cubic spline model showed a significant nonlinear association between IGF-1 (*P* for non-linearity = 1.1E-05; Fig. 3) and risk of dementia, with increases in the gradient of risk at around 18 nmol/. As can be seen from the nonlinear relationship between testosterone (*P* for non-linearity = 0.006; Fig. 3) and dementia, lower testosterone level is more deleterious to dementia events.

After rigorous adjustment for plenty of covariates (“Methods”), a variety of markers of immune and metabolic homeostasis were still significantly associated with an increased risk of incident dementia (Fig. 2). Consistent with previous studies linking dementia to insulin resistance and diabetes mellitus [14], high glucose (HR 1.093, 95% CI: 1.045–1.143, *p* = 0.003) and HbA1c (HR 1.017, 95% CI: 1.009–1.025, *p* = 0.001) levels were associated with increased dementia incidence. Lower LDL (HR 0.918, 95% CI: 0.872–0.965, *p* = 0.025) and triglycerides (HR 0.924, 95% CI: 0.882–0.967, *p* = 0.019) concentrations at baseline were associated with an increased risk of dementia, and similar triglycerides paradoxes were found in 2019 [15]. Moreover, we discovered strong U-shaped associations (Fig. 3) of C−reactive protein (CRP, *P* for non-linearity = 5.5E-09), and HbA1c (*P* for non-linearity = 0.007) with the risk of incident dementia, with nadirs at 2.5 mg/L, 34 mmol/mol, respectively.

Among liver function (Fig. 2), individuals with lower liver enzyme alanine aminotransferase (ALT, HR 0.990, 95% CI: 0.986–0.995, *p* = 2.6E-04) have a higher dementia incidence. Similar relationships were also observed by Kwangsik [16] and Yifei [8]. The cubic spline model showed a significant nonlinear J-shaped association between ALT and risk of dementia (*P* for non-linearity = 0.007; Fig. 3), reaching the lowest risk at around 30 U/L rapidly and then becoming flat thereafter, which also proved the role of decreased ALT in promoting dementia.

Finally, of the six markers classified as the renal function category, lower urate (HR 0.998, 95% CI: 0.998–0.999, *p* = 1.9E-06, Fig. 2) level, was associated with an increased risk of incident dementia. We also found strong non-linear association of creatinine (*P* for non-linearity = 0.009; Fig. 3), cystatin C (*P* for non-linearity = 0.028), urate (*P* for non-linearity = 0.007) and urea (*P* for non-linearity = 5.0E-09) with dementia. The other three markers, lower phosphate (HR 0.838, 95% CI: 0.642–1.092, *p* = 1.000), lower total protein (HR 0.998, 95% CI: 0.988–1.008, *p* = 1.000), and lower creatinine (HR 0.997, 95% CI: 0.994–1.000, *p* = 1.000), all trend towards a higher risk of incident dementia, which may imply a lack of energy, though the associations were not statistically significant.

**Fig. 4:**
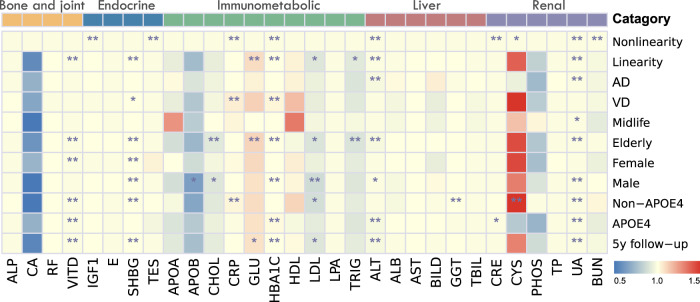
Heatmap to overview the associations of serum laboratory tests with dementia risks. Heatmap overview the results of restricted cubic spline models, Cox models in whole cohort for dementia and two dementia subtypes (Alzheimer disease and vascular dementia), sensitivity analysis (Cox models in midlife, elderly, female, male, *APOE ε4* non-carrier, *APOE ε4* carrier and follow-up time more than 5 years cohort, respectively). *p < 0.05, **p < 0.01. ALP Alkaline phosphatase, CA Calcium, RF Rheumatoid factor, VITD Vitamin D, IGF1 IGF-1; E Oestradiol, SHBG Sex hormone-binding globulin, TES Testosterone, APOA Apolipoprotein A, APOB Apolipoprotein B, CHOL Cholesterol, CRP C-reactive protein, GLU Glucose, HBA1C Glycated haemoglobin, HDL HDL cholesterol, LDL LDL cholesterol, LPA Lipoprotein A, TRIG Triglycerides, ALT Alanine aminotransferase, ALB Albumin, AST Aspartate aminotransferase, BILD Direct bilirubin, GGT Gamma glutamyltransferase, TBIL Total bilirubin, CRE Creatinine, CYS Cystatin C, PHOS Phosphate, TP Total protein, UA Urate, BUN Urea, *APOE4* apolipoprotein E ε4.

In sensitivity analyses, we found that HRs were generally in the same direction using stratified analyses based on dementia risk factors and putative effect modifiers such as participants’ age, sex, and *APOE* ${\varepsilon}$*4* carrier status (Fig. 4; Supplementary Tables 3–5). Summarizing the results of interaction analyses, we found no interactive effects of laboratory indicators with sex or age for the risk of dementia. The associations of creatinine, testosterone, and ALT with the risk of dementia were stronger among *APOE* ϵ*4* carriers (*P* for interaction = 0.0001, 0.0001, and 0.0178) while gamma glutamyltransferase (GGT), cystatin C, total cholesterol, and LDL were stronger among *APOE* ϵ*4* non-carriers (*P* for interaction = 0.0001, 0.0004, 0.0042, and 0.0088).

After excluding who experienced incident dementia event during the first 5 years of follow up (Supplementary Table 6), some linear associations of serum laboratory tests with the risk of dementia remained significant, such as SHBG, glucose, HbA1c, LDL, ALT, urate and vitamin D.

In sensitivity analyses for dementia subtypes, significant associations of low ALT (HR 0.987, 95% CI: 0.981–0.994, *p* = 0.006), urate (HR 0.998, 95% CI: 0.997–0.999, *p* = 0.004) concentrations with AD were described in Fig. 4 and Supplementary Table 7. As for VD, high levels of SHBG (HR 1.007, 95% CI: 1.003–1.011, *p* = 0.016), CRP (HR 1.052, 95% CI: 1.027–1.078, *p* = 0.001), and HbA1c (HR 1.039, 95% CI: 1.024–1.055, *p* = 1.1E-05) were significantly associated with it.

The final sensitivity analyses showed substantial change only for indicators among “immunometabolic” category, where the significance level and effect size of glucose, HbA1c, LDL, and triglycerides were reduced after additionally excluding participants who have pre-existing unbalanced comorbidities (Supplementary Table 9) in this category after Bonferroni correction.

## Discussion

In this study of 407,190 UKB participants in the community-based cohort over a median follow-up time of 9.19 years, we conducted a broad search to investigate the linear and nonlinear associations between serum laboratory tests and incident dementia. The 30 serum signatures detected were divided into several categories to reflect potential relationships with specific types of systemic dysfunction. Herein, we found multiple associations between incident dementia and markers representing specific dysfunction, including vitamin D deficiency, GH-IGF-1 axis disorders, bioactive sex hormone deficiency, liver and renal abnormalities, oxidation, lipids dysregulation, insulin resistance, and immune dysbiosis. Using the power of the hundreds of thousands of participants who were not demented at baseline in the UK Biobank, a large cohort with detailed sociodemographic and lifestyle information, we were able to maintain methodological consistency across markers, extensively correct for potential confounders, and perform multiple sensitivity analyses. To our knowledge, this is the first study utilizing full-scale serum laboratory tests to incident dementia.

In the analysis, vitamin D has shown to be a critical marker for predicting dementia incidents. One explanation of this relationship is the direct involvement of vitamin D in the pathogenesis of dementia [17–19], including the inhibition of neuroprotective functions such as anti-inflammatory, antioxidant effect on neurons, and the reduction of amyloid-β (Aβ) and phosphorylated tau [17]. However, the results of further randomized clinical trials regarding the benefits of vitamin D supplementation in dementia remain inconclusive [18].

Significant correlations between multiple endocrine dysregulations and the risk of dementia were also identified. The results of lower testosterone levels have shown alignment with the previous investigation, in which the administration of testosterone replacement therapy (TRT) has demonstrated its ability to improve cognitive function for older men in randomized clinical trials as a preventative treatment against AD and dementia [20–22]. SHBG, a secreted protein that plays a vital role in balancing bioactive sex hormones, has also demonstrated consistency with prior studies, in which higher levels of SHBG are associated with greater risks of dementia [23]. Therefore, bioactive sex hormone deficiency, reflected by levels of SHBG and testosterone, could potentially be used to predict dementia incidents [24]. IGF-1 was another profound endocrinal marker observed in our analysis. Evidence has indicated that individuals with low IGF-1 could fail to exert neuroprotection against oxidative stress and neuroinflammation in the brain as a neurotrophic factor [25], and thus the activity of the GH-IGF-1 axis declines progressively with advancing age may be mechanistically involved in AD pathogenesis [26, 27]. As for IGF-1 dysregulation in a high direction, Zhang et al. suggested that IGF‐1 interacts with age to modify hazards for dementia [28]. While IGF‐1 can protect against dementia in younger individuals, it is conversely associated with an increased risk of dementia in the elderly. One possible mechanism is the inhibition of autophagy, which prevents core processes of repair and maintenance in the nervous system for older individuals [28].

Reduced liver synthesis and metabolic function, indexed by decreased ALT levels [16], have shown to be linked with higher dementia risks. As the key enzyme catalyzing the process of pyruvate formation from alanine and α-ketoglutarate, reduced ALT associated with lower availability of pyruvate and may be related to decreased gluconeogenesis in the liver, therefore result in lower levels of glucose available as energy source to various tissues, especially in the orbitofrontal cortex and temporal lobes, brain regions implicated in executive and memory function [8, 16]. Furthermore, liver dysfunctions might lead to decreased liver-mediated clearance of peripheral Aβ, which may be a mechanism underlying dementia [29, 30].

Multiple markers reflecting renal insufficiency were associated with increased dementia incidence, such as elevated cystatin C, creatinine and urea, as demonstrated in the U-shaped association. While individuals with chronic kidney disease have been proved at substantially higher risk for cognitive impairment [31–33], this study is the first and largest to show a longitudinal association between these markers and dementia comprehensively. Nevertheless, a disturbance in Aβ clearance by the kidney might explain the association between renal dysfunction and impaired cognition [30, 34, 35]. Notably, the association with low level is difficult to interpret, as it also results from liver dysfunction or malnutrition, which is perhaps responsible for the association. As for urate, several studies also found a trend toward increased risk with lower urate concentration, and they suggest this may be explained by the fact that urate is a major antioxidant in the human body. A low concentration of it might be associated with more oxidative stress and thereby contribute to the development of dementia [36, 37].

Manifold dysregulated metabolic and immune pathways were associated with elevated risk of dementia, including insulin resistance, lipids dysregulation, and immune dysbiosis. The association of dementia with high glucose and HbA1c levels are intimately linked to insulin resistance, it is not surprising since downregulation of brain insulin receptors has been shown to promote tau phosphorylation, synaptic impairments, and memory loss [38, 39]. As for inflammatory markers such as CRP, an important signaling molecule in inflammation that has effects on the brains or the periphery of people with dementia [40], which can explain why high CRP levels may be harmful. In addition, two prospective cohort studies previously examined change in CRP level with dementia and cognition [41, 42], which suggesting low plasma CRP level were also associated with high risk of dementia and cognitive impairment. Mechanistically, it possibly because low CRP levels is a marker of poor immune function, which can lead to decreased phagocytosis of Aβ by microglia, decreased opsonization, and decreased activation of the complement system, thus resulting in a less efficient clearance of Aβ [41, 42]. Moreover, we find a significant association between dementia incidence and lower lipid fractions (triglycerides and LDL) levels, in contrast to some prior studies [7]. Several reasons might account for the apparent discrepancies between our findings and the others. Firstly, a curvilinear relationship between these three types of lipids and dementia possibly because low lipid concentrations reflect a lack of energy. Secondly, this bias might have been caused by unbalanced comorbidities, thus the significance level and effect size of the lipid relationship decreased after additional exclusion of participants with corresponding diseases.

The main strength of our study is that we use longitudinal rather than cross-sectional analyses to determine the associations of serum laboratory tests with incident dementia. Other strengths include the large sample size, long follow-up, extensive measurement of covariates, and that our diagnoses were based on hospital inpatient or primary care records.

Although there are important discoveries revealed by these studies, there are also limitations. First, potential confounding may still influence our results even though we have performed extensive covariate correction; Second, there may be some unaccounted for covariates that are not included, and those that are included may not be fully reliable, particularly for self-reported measures of drug use; Third, our 30 blood markers contain only a fraction of those ever tested in the UKB and may not be the best markers. Meanwhile, most of these indicators are already widely used in clinics, which makes it easier to translate our findings into clinically useful screening tools.

In conclusion, we find that markers of vitamin D deficiency, GH-IGF-1 axis disorders, bioactive sex hormone deficiency, reduced liver function, renal abnormalities, oxidation, insulin resistance, immune dysbiosis, and lipids dysregulation are associated with incident dementia in a large population-based cohort, supporting additional potential biomarkers and biological pathways to improve early detection and prevention of dementia patients. Our results advocate interventions to supplement vitamin D, raise growth hormone and testosterone levels, protect hepatic and renal function, maintain redox homeostasis, enhance immunity, and improve glycemic control and lipid profile to prevent dementia. Importantly, our study is valuable for the prediction of dementia, especially in the large-scale screening or primary care setting. Therefore, we suggest that when these blood biomarkers are at deviations from normal concentrations, more attention should be focused on cognition.

## Supplementary information

Supplementary material

## Data Availability

All code used for data preparation and analysis are available upon request.
