# Supplementary material for: Serum clinical laboratory tests and risk of incident dementia: a prospective cohort study of 407,190 individuals
Source: Transl Psychiatry. 2022 Aug 4;12:312. doi: 10.1038/s41398-022-02082-x (PMC9352702; doi:10.1038/s41398-022-02082-x)
Supplement: Supplementary file 1 — Supplementary material [file 41398_2022_2082_MOESM1_ESM.docx]

[Supplemental Table 1. Baseline characters of 30 serum clinical laboratory tests by incident dementia status. 2](#_Toc105018706)

[Supplemental Table 2. Linear associations between serum laboratory test and risk of incident dementia. 4](#_Toc105018707)

[Supplemental Table 3. Association between serum laboratory test and incident dementia stratified by age. 6](#_Toc105018708)

[Supplemental Table 4. Association between serum laboratory test and incident dementia stratified by sex. 8](#_Toc105018709)

[Supplemental Table 5. Association between serum laboratory test and incident dementia stratified by *APOE ε4* carrier status. 10](#_Toc105018710)

[Supplemental Table 6. Association between serum laboratory test and incident dementia after 5 years of follow-up. 12](#_Toc105018711)

[Supplemental Table 7. Association between serum laboratory test and incident Alzheimer's disease. 14](#_Toc105018712)

[Supplemental Table 8. Association between serum laboratory test and incident vascular dementia. 16](#_Toc105018713)

[Supplemental Table 9. Linear and nonlinear associations between serum laboratory test and risk of incident dementia after excluding participants with comorbidities. 18](#_Toc105018714)

[Supplemental Figure1. Pearson correlation coefficients for each pair of serum laboratory tests. 21](#_Toc105018715)

[Supplemental Figure2. Continuation of Figure 2. 22](#_Toc105018716)

## Supplemental Table 1. Baseline characters of 30 serum clinical laboratory tests by incident dementia status.

| **Characteristics** | **Overall** | **No incident dementia** | **Incident dementia** | **P value** |
| --- | --- | --- | --- | --- |
| Alkaline phosphatase, mean (SD), U/L | 83.40 (22.78) | 83.36 (22.76) | 86.75 (23.90) | <0.001 |
| Calcium, mean (SD), mmol/L | 2.38 (0.09) | 2.38 (0.09) | 2.38 (0.10) | 0.007 |
| Rheumatoid factor, mean (SD), IU/ml | 23.48 (17.47) | 23.46 (17.45) | 24.43 (18.52) | 0.23 |
| Vitamin D, mean (SD), nmol/L | 49.34 (20.72) | 49.36 (20.72) | 47.98 (20.98) | <0.001 |
| IGF-1, mean (SD), nmol/L | 21.22 (5.54) | 21.24 (5.53) | 20.11 (5.79) | <0.001 |
| Oestradiol, mean (SD), pmol/L | 429.81 (322.47) | 430.71 (322.90) | 286.97 (197.32) | <0.001 |
| SHBG, mean (SD), nmol/L | 50.92 (25.43) | 50.89 (25.43) | 52.90 (25.06) | <0.001 |
| Testosterone, mean (SD), nmol/L | 6.57 (6.02) | 6.56 (6.02) | 7.33 (6.00) | <0.001 |
| Apolipoprotein A, mean (SD), g/L | 1.54 (0.27) | 1.54 (0.27) | 1.52 (0.28) | <0.001 |
| Apolipoprotein B, mean (SD), g/L | 1.03 (0.24) | 1.03 (0.24) | 1.01 (0.25) | <0.001 |
| Cholesterol, mean (SD), mmol/L | 5.70 (1.14) | 5.70 (1.14) | 5.49 (1.28) | <0.001 |
| C-reactive protein, mean (SD), mg/L | 2.33 (2.84) | 2.33 (2.83) | 2.51 (3.10) | <0.001 |
| Glucose, mean (SD), mmol/L | 5.03 (0.78) | 5.03 (0.78) | 5.21 (0.93) | <0.001 |
| Glycated haemoglobin, mean (SD), mmol/mol | 35.66 (4.88) | 35.64 (4.86) | 37.47 (5.96) | <0.001 |
| HDL cholesterol, mean (SD), mmol/L | 1.44 (0.38) | 1.44 (0.38) | 1.41 (0.39) | <0.001 |
| LDL direct, mean (SD), mmol/L | 3.56 (0.87) | 3.56 (0.87) | 3.41 (0.95) | <0.001 |
| Lipoprotein A, mean (SD), nmol/L | 44.09 (49.32) | 44.09 (49.32) | 44.07 (49.64) | 0.979 |
| Triglycerides, mean (SD), mmol/L | 1.73 (0.92) | 1.73 (0.92) | 1.75 (0.90) | 0.071 |
| Alanine aminotransferase, mean (SD), U/L | 22.94 (11.10) | 22.95 (11.11) | 21.99 (10.05) | <0.001 |
| Albumin, mean (SD), g/L | 45.17 (2.59) | 45.18 (2.59) | 44.72 (2.63) | <0.001 |
| Aspartate aminotransferase, mean (SD), U/L | 25.78 (7.30) | 25.77 (7.29) | 26.72 (7.65) | <0.001 |
| Direct bilirubin, mean (SD), umol/L | 1.80 (0.70) | 1.80 (0.70) | 1.84 (0.72) | <0.001 |
| Gamma glutamyltransferase, mean (SD), U/L | 35.04 (27.10) | 35.00 (27.06) | 38.35 (30.21) | <0.001 |
| Total bilirubin, mean (SD), umol/L | 8.88 (3.67) | 8.88 (3.67) | 8.94 (3.69) | 0.205 |
| Creatinine, mean (SD), umol/L | 71.96 (14.53) | 71.93 (14.50) | 73.85 (16.40) | <0.001 |
| Cystatin C, mean (SD), mg/L | 0.91 (0.15) | 0.91 (0.14) | 0.97 (0.17) | <0.001 |
| Phosphate, mean (SD), mmol/L | 1.16 (0.16) | 1.16 (0.16) | 1.16 (0.16) | 0.422 |
| Total protein, mean (SD), g/L | 72.32 (4.01) | 72.32 (4.00) | 72.09 (4.17) | <0.001 |
| Urate, mean (SD), umol/L | 310.00 (80.17) | 309.90 (80.13) | 317.38 (82.49) | <0.001 |
| Urea, mean (SD), mmol/L | 5.42 (1.29) | 5.42 (1.29) | 5.68 (1.49) | <0.001 |

Values are mean (standard deviation) or numbers (percentage). P-values are derived using either Student’s t-test or Chi-square test.

## Supplemental Table 2. Linear associations between serum laboratory test and risk of incident dementia.

| Dementia | Model 1*^a^* | | |  | Model 2*^b^* | | |
| --- | --- | --- | --- | --- | --- | --- | --- |
|  | Event No./Total No. | HR ( 95% CI ) | P value (Bonferroni) |  | Event No./Total No. | HR ( 95% CI ) | P value (Bonferroni) |
| Alkaline phosphatase | 4513/351000 | 1.002 ( 1.000, 1.003 ) | 0.3087 |  | 2802/281336 | 0.999 ( 0.998, 1.001 ) | 1.0000 |
| Calcium | 4148/321630 | 0.747 ( 0.535, 1.045 ) | 1.0000 |  | 2575/257512 | 0.574 ( 0.375, 0.880 ) | 0.3278 |
| Rheumatoid factor | 438/31423 | 1.000 ( 0.995, 1.005 ) | 1.0000 |  | 271/24828 | 1.003 ( 0.997, 1.010 ) | 1.0000 |
| Vitamin D | 4324/336366 | 0.993 ( 0.991, 0.994 ) | **0.0000** |  | 2679/269868 | 0.994 ( 0.993, 0.996 ) | **0.0000** |
| IGF-1 | 4523/349849 | 0.999 ( 0.994, 1.005 ) | 1.0000 |  | 2802/280306 | 1.007 ( 1.000, 1.014 ) | 1.0000 |
| Oestradiol | 330/53368 | 1.000 ( 1.000, 1.001 ) | 1.0000 |  | 203/47206 | 1.000 ( 0.999, 1.000 ) | 1.0000 |
| SHBG | 4099/317294 | 1.004 ( 1.002, 1.005 ) | **0.0000** |  | 2544/253909 | 1.004 ( 1.003, 1.006 ) | **0.0001** |
| Testosterone | 4005/318047 | 0.993 ( 0.982, 1.004 ) | 1.0000 |  | 2495/255892 | 0.999 ( 0.985, 1.013 ) | 1.0000 |
| Apolipoprotein A | 4127/320538 | 0.869 ( 0.767, 0.984 ) | 0.8086 |  | 2565/256564 | 0.944 ( 0.803, 1.109 ) | 1.0000 |
| Apolipoprotein B | 4517/350529 | 0.579 ( 0.512, 0.655 ) | **0.0000** |  | 2800/280840 | 0.756 ( 0.632, 0.903 ) | 0.0603 |
| Cholesterol | 4538/352077 | 0.891 ( 0.869, 0.914 ) | **0.0000** |  | 2814/282078 | 0.942 ( 0.907, 0.978 ) | 0.0619 |
| C-reactive protein | 4472/347509 | 1.015 ( 1.005, 1.025 ) | 0.0878 |  | 2772/278778 | 1.019 ( 1.005, 1.033 ) | 0.1801 |
| Glucose | 4038/318466 | 1.126 ( 1.088, 1.165 ) | **0.0000** |  | 2519/255276 | 1.093 ( 1.045, 1.143 ) | **0.0030** |
| Glycated haemoglobin | 4417/348384 | 1.024 ( 1.019, 1.030 ) | **0.0000** |  | 2743/279450 | 1.017 ( 1.009, 1.025 ) | **0.0008** |
| HDL cholesterol | 4149/321809 | 0.976 ( 0.892, 1.067 ) | 1.0000 |  | 2573/257612 | 1.042 ( 0.924, 1.174 ) | 1.0000 |
| LDL direct | 4527/351445 | 0.853 ( 0.825, 0.881 ) | **0.0000** |  | 2810/281573 | 0.918 ( 0.872, 0.965 ) | **0.0252** |
| Lipoprotein A | 3579/280012 | 1.000 ( 0.999, 1.000 ) | 1.0000 |  | 2243/224639 | 0.999 ( 0.999, 1.000 ) | 1.0000 |
| Triglycerides | 4509/349473 | 0.940 ( 0.909, 0.973 ) | **0.0109** |  | 2800/280081 | 0.924 ( 0.882, 0.967 ) | **0.0187** |
| Alanine aminotransferase | 4510/349468 | 0.993 ( 0.989, 0.996 ) | **0.0001** |  | 2800/279999 | 0.990 ( 0.986, 0.995 ) | **0.0003** |
| Albumin | 4148/322208 | 0.991 ( 0.979, 1.003 ) | 1.0000 |  | 2578/257972 | 0.997 ( 0.981, 1.012 ) | 1.0000 |
| Aspartate aminotransferase | 4478/348673 | 1.006 ( 1.002, 1.010 ) | 0.0829 |  | 2774/279452 | 1.004 ( 0.999, 1.010 ) | 1.0000 |
| Direct bilirubin | 3918/296926 | 1.028 ( 0.983, 1.076 ) | 1.0000 |  | 2469/238960 | 1.005 ( 0.949, 1.064 ) | 1.0000 |
| Gamma glutamyltransferase | 4470/348817 | 1.002 ( 1.001, 1.003 ) | **0.0003** |  | 2774/279670 | 1.002 ( 1.001, 1.003 ) | 0.1265 |
| Total bilirubin | 4496/347400 | 0.992 ( 0.984, 1.001 ) | 1.0000 |  | 2783/278195 | 0.995 ( 0.984, 1.005 ) | 1.0000 |
| Creatinine | 4492/351135 | 0.998 ( 0.996, 1.001 ) | 1.0000 |  | 2793/281466 | 0.997 ( 0.994, 1.000 ) | 1.0000 |
| Cystatin C | 4456/350484 | 1.762 ( 1.455, 2.134 ) | **0.0000** |  | 2775/281146 | 1.394 ( 1.071, 1.816 ) | 0.4083 |
| Phosphate | 4152/321794 | 1.058 ( 0.859, 1.303 ) | 1.0000 |  | 2579/257597 | 0.838 ( 0.642, 1.092 ) | 1.0000 |
| Total protein | 4156/321811 | 1.002 ( 0.994, 1.010 ) | 1.0000 |  | 2580/257664 | 0.998 ( 0.988, 1.008 ) | 1.0000 |
| Urate | 4527/351572 | 0.999 ( 0.998, 0.999 ) | **0.0000** |  | 2808/281710 | 0.998 ( 0.998, 0.999 ) | **0.0000** |
| Urea | 4487/350760 | 0.967 ( 0.945, 0.989 ) | 0.1140 |  | 2791/281222 | 0.983 ( 0.955, 1.012 ) | 1.0000 |

***^a^***Model 1 adjusted for demographic and temporal variables.

***^b^***Model 2 additionally adjusted for socioeconomic, lifestyle and medication variables.

P value column in bold indicates statistical significance at a Bonferroni adjusted p value < 0.05.

Abbreviations: CI, confidence interval; HR, hazard ratio;

## Supplemental Table 3. Association between serum laboratory test and incident dementia stratified by age.

| Dementia | Model 2*^a^* | | | | | | | |
| --- | --- | --- | --- | --- | --- | --- | --- | --- |
|  | Event No./Total No. | HR ( 95% CI ) | P value (Bonferroni) |  | Event No./Total No. | HR ( 95% CI ) | P value (Bonferroni) | P value for interaction |
|  | **Age≤60** | | |  | **Age>60** | | | |
| Alkaline phosphatase | 510/178496 | 1.000 ( 0.996, 1.004 ) | 1.0000 |  | 2292/102840 | 0.999 ( 0.997, 1.001 ) | 1.0000 | 1.0000 |
| Calcium | 471/163079 | 0.478 ( 0.173, 1.317 ) | 1.0000 |  | 2104/94433 | 0.609 ( 0.380, 0.976 ) | 1.0000 | 1.0000 |
| Rheumatoid factor | 46/14511 | 1.012 ( 0.999, 1.026 ) | 1.0000 |  | 225/10317 | 1.001 ( 0.994, 1.008 ) | 1.0000 | 1.0000 |
| Vitamin D | 490/171780 | 0.995 ( 0.990, 0.999 ) | 0.7170 |  | 2189/98088 | 0.994 ( 0.992, 0.997 ) | **0.0000** | 1.0000 |
| IGF-1 | 512/177738 | 1.001 ( 0.985, 1.018 ) | 1.0000 |  | 2290/102568 | 1.008 ( 1.000, 1.016 ) | 1.0000 | 1.0000 |
| Oestradiol | 65/41122 | 0.999 ( 0.998, 1.000 ) | 1.0000 |  | 138/6084 | 1.000 ( 0.999, 1.001 ) | 1.0000 | 1.0000 |
| SHBG | 466/160463 | 1.006 ( 1.002, 1.010 ) | 0.1005 |  | 2078/93446 | 1.004 ( 1.002, 1.006 ) | **0.0069** | 1.0000 |
| Testosterone | 470/164438 | 0.994 ( 0.961, 1.028 ) | 1.0000 |  | 2025/91454 | 1.000 ( 0.984, 1.015 ) | 1.0000 | 1.0000 |
| Apolipoprotein A | 468/162505 | 1.278 ( 0.881, 1.853 ) | 1.0000 |  | 2097/94059 | 0.882 ( 0.737, 1.055 ) | 1.0000 | 1.0000 |
| Apolipoprotein B | 511/178039 | 0.813 ( 0.545, 1.215 ) | 1.0000 |  | 2289/102801 | 0.735 ( 0.603, 0.898 ) | 0.0754 | 1.0000 |
| Cholesterol | 515/178922 | 1.009 ( 0.926, 1.099 ) | 1.0000 |  | 2299/103156 | 0.924 ( 0.886, 0.965 ) | **0.0096** | 1.0000 |
| C-reactive protein | 508/177037 | 1.032 ( 1.001, 1.064 ) | 1.0000 |  | 2264/101741 | 1.016 ( 1.001, 1.031 ) | 1.0000 | 1.0000 |
| Glucose | 459/161730 | 1.001 ( 0.893, 1.121 ) | 1.0000 |  | 2060/93546 | 1.112 ( 1.060, 1.168 ) | **0.0005** | 1.0000 |
| Glycated haemoglobin | 493/177370 | 1.015 ( 0.996, 1.034 ) | 1.0000 |  | 2250/102080 | 1.017 ( 1.009, 1.026 ) | **0.0028** | 1.0000 |
| HDL cholesterol | 469/163114 | 1.331 ( 1.010, 1.753 ) | 1.0000 |  | 2104/94498 | 0.988 ( 0.865, 1.127 ) | 1.0000 | 1.0000 |
| LDL direct | 512/178587 | 0.972 ( 0.868, 1.088 ) | 1.0000 |  | 2298/102986 | 0.903 ( 0.853, 0.955 ) | **0.0118** | 1.0000 |
| Lipoprotein A | 409/142567 | 0.999 ( 0.997, 1.001 ) | 1.0000 |  | 1834/82072 | 1.000 ( 0.999, 1.001 ) | 1.0000 | 1.0000 |
| Triglycerides | 510/177444 | 0.981 ( 0.889, 1.082 ) | 1.0000 |  | 2290/102637 | 0.907 ( 0.862, 0.955 ) | **0.0065** | 1.0000 |
| Alanine aminotransferase | 507/177314 | 0.994 ( 0.986, 1.003 ) | 1.0000 |  | 2293/102685 | 0.989 ( 0.984, 0.994 ) | **0.0003** | 1.0000 |
| Albumin | 468/163328 | 0.983 ( 0.948, 1.019 ) | 1.0000 |  | 2110/94644 | 1.000 ( 0.983, 1.018 ) | 1.0000 | 1.0000 |
| Aspartate aminotransferase | 497/177163 | 1.012 ( 1.001, 1.024 ) | 0.9936 |  | 2277/102289 | 1.002 ( 0.996, 1.008 ) | 1.0000 | 1.0000 |
| Direct bilirubin | 439/150747 | 0.963 ( 0.836, 1.108 ) | 1.0000 |  | 2030/88213 | 1.013 ( 0.951, 1.079 ) | 1.0000 | 1.0000 |
| Gamma glutamyltransferase | 498/177393 | 1.003 ( 1.001, 1.006 ) | 0.4806 |  | 2276/102277 | 1.002 ( 1.000, 1.003 ) | 1.0000 | 1.0000 |
| Total bilirubin | 510/176275 | 0.985 ( 0.960, 1.011 ) | 1.0000 |  | 2273/101920 | 0.997 ( 0.985, 1.008 ) | 1.0000 | 1.0000 |
| Creatinine | 509/178642 | 0.991 ( 0.984, 0.999 ) | 0.8664 |  | 2284/102824 | 0.998 ( 0.995, 1.001 ) | 1.0000 | 1.0000 |
| Cystatin C | 506/178596 | 1.163 ( 0.582, 2.326 ) | 1.0000 |  | 2269/102550 | 1.448 ( 1.088, 1.927 ) | 0.3331 | 1.0000 |
| Phosphate | 469/163068 | 1.035 ( 0.564, 1.901 ) | 1.0000 |  | 2110/94529 | 0.799 ( 0.595, 1.073 ) | 1.0000 | 1.0000 |
| Total protein | 470/163131 | 0.991 ( 0.969, 1.015 ) | 1.0000 |  | 2110/94533 | 0.999 ( 0.989, 1.010 ) | 1.0000 | 1.0000 |
| Urate | 511/178659 | 0.998 ( 0.996, 0.999 ) | **0.0140** |  | 2297/103051 | 0.999 ( 0.998, 0.999 ) | **0.0006** | 1.0000 |
| Urea | 509/178558 | 0.932 ( 0.867, 1.002 ) | 1.0000 |  | 2282/102664 | 0.994 ( 0.964, 1.026 ) | 1.0000 | 1.0000 |

***^a^***Model 2 adjusted for demographic, temporal, socioeconomic, lifestyle and medication variables.

P value column in bold indicates statistical significance at a Bonferroni adjusted p value < 0.05.

Abbreviations: CI, confidence interval; HR, hazard ratio;

## Supplemental Table 4. Association between serum laboratory test and incident dementia stratified by sex.

| Dementia | Model 2*^a^* | | | | | | | |
| --- | --- | --- | --- | --- | --- | --- | --- | --- |
|  | Event No./Total No. | HR ( 95% CI ) | P value (Bonferroni) |  | Event No./Total No. | HR ( 95% CI ) | P value (Bonferroni) | P value for interaction |
|  | **Female** | | |  | **Male** | | | |
| Alkaline phosphatase | 1271/151431 | 0.999 ( 0.996, 1.001 ) | 1.0000 |  | 1531/129905 | 1.000 ( 0.997, 1.002 ) | 1.0000 | 1.0000 |
| Calcium | 1159/137549 | 0.729 ( 0.396, 1.344 ) | 1.0000 |  | 1416/119963 | 0.452 ( 0.249, 0.821 ) | 0.2755 | 1.0000 |
| Rheumatoid factor | 130/13764 | 1.005 ( 0.996, 1.014 ) | 1.0000 |  | 141/11064 | 1.001 ( 0.992, 1.011 ) | 1.0000 | 1.0000 |
| Vitamin D | 1181/143591 | 0.992 ( 0.989, 0.995 ) | **0.0000** |  | 1498/126277 | 0.996 ( 0.994, 0.999 ) | 0.1383 | 1.0000 |
| IGF-1 | 1273/150936 | 0.998 ( 0.987, 1.008 ) | 1.0000 |  | 1529/129370 | 1.015 ( 1.005, 1.024 ) | 0.0956 | 0.5093 |
| Oestradiol | 86/36207 | 0.999 ( 0.998, 1.000 ) | 1.0000 |  | 117/10999 | 1.002 ( 1.000, 1.004 ) | 0.4358 | 0.3466 |
| SHBG | 1135/134708 | 1.004 ( 1.002, 1.007 ) | **0.0082** |  | 1409/119201 | 1.006 ( 1.003, 1.009 ) | **0.0091** | 1.0000 |
| Testosterone | 979/126981 | 1.040 ( 0.952, 1.136 ) | 1.0000 |  | 1516/128911 | 0.993 ( 0.979, 1.008 ) | 1.0000 | 1.0000 |
| Apolipoprotein A | 1148/136670 | 1.007 ( 0.803, 1.263 ) | 1.0000 |  | 1417/119894 | 0.889 ( 0.706, 1.120 ) | 1.0000 | 1.0000 |
| Apolipoprotein B | 1275/151448 | 0.870 ( 0.675, 1.122 ) | 1.0000 |  | 1525/129392 | 0.644 ( 0.501, 0.827 ) | **0.0168** | 1.0000 |
| Cholesterol | 1278/151844 | 0.971 ( 0.919, 1.026 ) | 1.0000 |  | 1536/130234 | 0.911 ( 0.864, 0.961 ) | **0.0192** | 1.0000 |
| C-reactive protein | 1262/150179 | 1.022 ( 1.002, 1.042 ) | 0.9286 |  | 1510/128599 | 1.015 ( 0.997, 1.035 ) | 1.0000 | 1.0000 |
| Glucose | 1143/136978 | 1.106 ( 1.031, 1.186 ) | 0.1395 |  | 1376/118298 | 1.088 ( 1.027, 1.154 ) | 0.1354 | 1.0000 |
| Glycated haemoglobin | 1248/151024 | 1.014 ( 1.001, 1.027 ) | 0.9659 |  | 1495/128426 | 1.019 ( 1.009, 1.029 ) | **0.0062** | 1.0000 |
| HDL cholesterol | 1158/137592 | 1.056 ( 0.896, 1.246 ) | 1.0000 |  | 1415/120020 | 1.038 ( 0.873, 1.235 ) | 1.0000 | 1.0000 |
| LDL direct | 1278/151600 | 0.955 ( 0.889, 1.026 ) | 1.0000 |  | 1532/129973 | 0.875 ( 0.815, 0.940 ) | **0.0081** | 1.0000 |
| Lipoprotein A | 1030/121426 | 1.000 ( 0.999, 1.001 ) | 1.0000 |  | 1213/103213 | 0.999 ( 0.998, 1.000 ) | 1.0000 | 1.0000 |
| Triglycerides | 1277/151470 | 0.934 ( 0.868, 1.005 ) | 1.0000 |  | 1523/128611 | 0.917 ( 0.865, 0.972 ) | 0.1067 | 1.0000 |
| Alanine aminotransferase | 1276/151239 | 0.990 ( 0.984, 0.997 ) | 0.1693 |  | 1524/128760 | 0.990 ( 0.985, 0.996 ) | **0.0160** | 1.0000 |
| Albumin | 1161/137943 | 1.013 ( 0.990, 1.037 ) | 1.0000 |  | 1417/120029 | 0.984 ( 0.964, 1.005 ) | 1.0000 | 1.0000 |
| Aspartate aminotransferase | 1268/150755 | 1.011 ( 1.003, 1.019 ) | 0.2362 |  | 1506/128697 | 1.000 ( 0.993, 1.007 ) | 1.0000 | 0.7175 |
| Direct bilirubin | 1032/118657 | 0.950 ( 0.854, 1.057 ) | 1.0000 |  | 1437/120303 | 1.029 ( 0.961, 1.102 ) | 1.0000 | 1.0000 |
| Gamma glutamyltransferase | 1267/151098 | 1.002 ( 1.000, 1.004 ) | 1.0000 |  | 1507/128572 | 1.002 ( 1.000, 1.004 ) | 0.6074 | 1.0000 |
| Total bilirubin | 1274/150612 | 0.996 ( 0.979, 1.014 ) | 1.0000 |  | 1509/127583 | 0.994 ( 0.980, 1.007 ) | 1.0000 | 1.0000 |
| Creatinine | 1277/151758 | 1.000 ( 0.995, 1.005 ) | 1.0000 |  | 1516/129708 | 0.995 ( 0.991, 0.999 ) | 0.2555 | 1.0000 |
| Cystatin C | 1266/151560 | 1.457 ( 0.976, 2.176 ) | 1.0000 |  | 1509/129586 | 1.328 ( 0.934, 1.887 ) | 1.0000 | 1.0000 |
| Phosphate | 1161/137712 | 0.760 ( 0.508, 1.139 ) | 1.0000 |  | 1418/119885 | 0.907 ( 0.635, 1.295 ) | 1.0000 | 1.0000 |
| Total protein | 1161/137772 | 1.004 ( 0.990, 1.019 ) | 1.0000 |  | 1419/119892 | 0.993 ( 0.980, 1.006 ) | 1.0000 | 1.0000 |
| Urate | 1277/151744 | 0.999 ( 0.998, 0.999 ) | 0.0631 |  | 1531/129966 | 0.998 ( 0.998, 0.999 ) | **0.0001** | 1.0000 |
| Urea | 1273/151589 | 0.956 ( 0.915, 0.999 ) | 1.0000 |  | 1518/129633 | 1.004 ( 0.966, 1.043 ) | 1.0000 | 1.0000 |

***^a^***Model 2 adjusted for demographic, temporal, socioeconomic, lifestyle and medication variables.

P value column in bold indicates statistical significance at a Bonferroni adjusted p value < 0.05.

Abbreviations: CI, confidence interval; HR, hazard ratio;

## Supplemental Table 5. Association between serum laboratory test and incident dementia stratified by *APOE ε4* carrier status.

| Dementia | Model 2*^a^* | | | | | | | |
| --- | --- | --- | --- | --- | --- | --- | --- | --- |
|  | Event No./Total No. | HR ( 95% CI ) | P value (Bonferroni) |  | Event No./Total No. | HR ( 95% CI ) | P value (Bonferroni) | P value for interaction |
|  | **APOE 𝜀4 non-carrier** | | |  | **APOE 𝜀4 carrier** | | | |
| Alkaline phosphatase | 1301/200466 | 1.002 ( 0.999, 1.004 ) | 1.0000 |  | 1501/80870 | 0.998 ( 0.995, 1.000 ) | 1.0000 | 1.0000 |
| Calcium | 1197/183511 | 0.447 ( 0.239, 0.837 ) | 0.3568 |  | 1378/74001 | 0.710 ( 0.396, 1.272 ) | 1.0000 | 1.0000 |
| Rheumatoid factor | 126/17825 | 1.003 ( 0.993, 1.012 ) | 1.0000 |  | 145/7003 | 1.003 ( 0.995, 1.012 ) | 1.0000 | 1.0000 |
| Vitamin D | 1259/193364 | 0.995 ( 0.992, 0.998 ) | **0.0085** |  | 1420/76504 | 0.995 ( 0.992, 0.997 ) | **0.0027** | 1.0000 |
| IGF-1 | 1301/199771 | 1.008 ( 0.998, 1.018 ) | 1.0000 |  | 1501/80535 | 1.006 ( 0.996, 1.016 ) | 1.0000 | 1.0000 |
| Oestradiol | 120/33614 | 0.999 ( 0.998, 1.001 ) | 1.0000 |  | 83/13592 | 1.000 ( 0.999, 1.001 ) | 1.0000 | 1.0000 |
| SHBG | 1178/180984 | 1.006 ( 1.003, 1.009 ) | **0.0005** |  | 1366/72925 | 1.003 ( 1.000, 1.006 ) | 0.5824 | 1.0000 |
| Testosterone | 1170/182422 | 1.020 ( 1.000, 1.040 ) | 1.0000 |  | 1325/73470 | 0.978 ( 0.958, 0.998 ) | 0.9844 | **0.0001** |
| Apolipoprotein A | 1195/182733 | 0.984 ( 0.779, 1.244 ) | 1.0000 |  | 1370/73831 | 0.906 ( 0.726, 1.132 ) | 1.0000 | 0.9545 |
| Apolipoprotein B | 1299/200124 | 0.671 ( 0.515, 0.875 ) | 0.0972 |  | 1501/80716 | 0.804 ( 0.632, 1.023 ) | 1.0000 | 0.1377 |
| Cholesterol | 1307/201031 | 0.921 ( 0.870, 0.974 ) | 0.1299 |  | 1507/81047 | 0.953 ( 0.905, 1.003 ) | 1.0000 | **0.0042** |
| C-reactive protein | 1285/198436 | 1.033 ( 1.016, 1.051 ) | **0.0052** |  | 1487/80342 | 0.997 ( 0.976, 1.019 ) | 1.0000 | 0.1641 |
| Glucose | 1165/181885 | 1.096 ( 1.030, 1.167 ) | 0.1189 |  | 1354/73391 | 1.087 ( 1.019, 1.158 ) | 0.3234 | 1.0000 |
| Glycated haemoglobin | 1257/199173 | 1.009 ( 0.998, 1.021 ) | 1.0000 |  | 1486/80277 | 1.023 ( 1.012, 1.034 ) | **0.0008** | 1.0000 |
| HDL cholesterol | 1199/183533 | 1.112 ( 0.935, 1.323 ) | 1.0000 |  | 1374/74079 | 0.982 ( 0.833, 1.157 ) | 1.0000 | 1.0000 |
| LDL direct | 1302/200665 | 0.881 ( 0.818, 0.950 ) | **0.0301** |  | 1508/80908 | 0.938 ( 0.876, 1.004 ) | 1.0000 | **0.0088** |
| Lipoprotein A | 1035/160091 | 1.000 ( 0.998, 1.001 ) | 1.0000 |  | 1208/64548 | 0.999 ( 0.998, 1.001 ) | 1.0000 | 1.0000 |
| Triglycerides | 1298/199755 | 0.936 ( 0.876, 1.000 ) | 1.0000 |  | 1502/80326 | 0.913 ( 0.858, 0.973 ) | 0.1459 | 1.0000 |
| Alanine aminotransferase | 1294/199437 | 0.992 ( 0.987, 0.998 ) | 0.3236 |  | 1506/80562 | 0.988 ( 0.982, 0.994 ) | **0.0028** | **0.0178** |
| Albumin | 1197/183822 | 0.986 ( 0.964, 1.008 ) | 1.0000 |  | 1381/74150 | 1.006 ( 0.985, 1.027 ) | 1.0000 | 1.0000 |
| Aspartate aminotransferase | 1281/199103 | 1.002 ( 0.994, 1.009 ) | 1.0000 |  | 1493/80349 | 1.006 ( 0.999, 1.014 ) | 1.0000 | 1.0000 |
| Direct bilirubin | 1159/170909 | 0.994 ( 0.914, 1.081 ) | 1.0000 |  | 1310/68051 | 1.013 ( 0.936, 1.096 ) | 1.0000 | 1.0000 |
| Gamma glutamyltransferase | 1283/199252 | 1.004 ( 1.002, 1.006 ) | **0.0004** |  | 1491/80418 | 1.000 ( 0.998, 1.002 ) | 1.0000 | **0.0001** |
| Total bilirubin | 1288/198309 | 0.991 ( 0.975, 1.007 ) | 1.0000 |  | 1495/79886 | 0.997 ( 0.983, 1.012 ) | 1.0000 | 1.0000 |
| Creatinine | 1292/200601 | 1.001 ( 0.997, 1.005 ) | 1.0000 |  | 1501/80865 | 0.993 ( 0.989, 0.997 ) | **0.0278** | **0.0001** |
| Cystatin C | 1283/200342 | 2.440 ( 1.685, 3.534 ) | **0.0001** |  | 1492/80804 | 0.809 ( 0.556, 1.179 ) | 1.0000 | **0.0004** |
| Phosphate | 1198/183544 | 0.996 ( 0.676, 1.467 ) | 1.0000 |  | 1381/74053 | 0.721 ( 0.501, 1.037 ) | 1.0000 | 1.0000 |
| Total protein | 1199/183594 | 0.999 ( 0.985, 1.013 ) | 1.0000 |  | 1381/74070 | 0.997 ( 0.984, 1.010 ) | 1.0000 | 1.0000 |
| Urate | 1302/200754 | 0.998 ( 0.998, 0.999 ) | **0.0034** |  | 1506/80956 | 0.999 ( 0.998, 0.999 ) | **0.0089** | 0.3474 |
| Urea | 1290/200389 | 1.032 ( 0.990, 1.076 ) | 1.0000 |  | 1501/80833 | 0.943 ( 0.905, 0.981 ) | 0.1181 | 0.0979 |

***^a^***Model 2 adjusted for demographic, temporal, socioeconomic, lifestyle and medication variables.

P value column in bold indicates statistical significance at a Bonferroni adjusted p value < 0.05.

Abbreviations: CI, confidence interval; HR, hazard ratio;

## Supplemental Table 6. Association between serum laboratory test and incident dementia after 5 years of follow-up.

| Dementia | Model 1*^a^* | | |  | Model 2*^b^* | | |
| --- | --- | --- | --- | --- | --- | --- | --- |
|  | Event No./Total No. | HR ( 95% CI ) | P value (Bonferroni) |  | Event No./Total No. | HR ( 95% CI ) | P value (Bonferroni) |
| Alkaline phosphatase | 3806/322355 | 1.001 ( 1.000, 1.002 ) | 1.0000 |  | 2374/258239 | 0.999 ( 0.997, 1.001 ) | 1.0000 |
| Calcium | 3488/295125 | 0.733 ( 0.509, 1.056 ) | 1.0000 |  | 2178/236155 | 0.558 ( 0.351, 0.888 ) | 0.4156 |
| Rheumatoid factor | 372/28791 | 1.001 ( 0.996, 1.007 ) | 1.0000 |  | 228/22791 | 1.005 ( 0.998, 1.011 ) | 1.0000 |
| Vitamin D | 3642/308809 | 0.994 ( 0.992, 0.995 ) | **0.0000** |  | 2272/247643 | 0.995 ( 0.993, 0.997 ) | **0.0003** |
| IGF-1 | 3807/321190 | 1.002 ( 0.996, 1.008 ) | 1.0000 |  | 2369/257208 | 1.009 ( 1.001, 1.017 ) | 0.7043 |
| Oestradiol | 266/48861 | 1.000 ( 0.999, 1.001 ) | 1.0000 |  | 169/43237 | 0.999 ( 0.999, 1.000 ) | 1.0000 |
| SHBG | 3445/291137 | 1.003 ( 1.002, 1.005 ) | **0.0008** |  | 2151/232845 | 1.004 ( 1.002, 1.006 ) | **0.0046** |
| Testosterone | 3360/291916 | 0.992 ( 0.980, 1.004 ) | 1.0000 |  | 2107/234799 | 0.998 ( 0.983, 1.014 ) | 1.0000 |
| Apolipoprotein A | 3469/294110 | 0.872 ( 0.761, 0.999 ) | 1.0000 |  | 2170/235287 | 0.925 ( 0.776, 1.102 ) | 1.0000 |
| Apolipoprotein B | 3802/321808 | 0.590 ( 0.515, 0.674 ) | **0.0000** |  | 2369/257699 | 0.762 ( 0.628, 0.925 ) | 0.1765 |
| Cholesterol | 3822/323219 | 0.895 ( 0.871, 0.920 ) | **0.0000** |  | 2382/258827 | 0.940 ( 0.901, 0.979 ) | 0.0983 |
| C-reactive protein | 3771/319217 | 1.011 ( 1.000, 1.022 ) | 1.0000 |  | 2347/255910 | 1.014 ( 0.999, 1.029 ) | 1.0000 |
| Glucose | 3395/292243 | 1.125 ( 1.085, 1.167 ) | **0.0000** |  | 2129/234102 | 1.085 ( 1.033, 1.139 ) | **0.0307** |
| Glycated haemoglobin | 3714/319894 | 1.024 ( 1.018, 1.030 ) | **0.0000** |  | 2306/256458 | 1.016 ( 1.008, 1.025 ) | **0.0060** |
| HDL cholesterol | 3489/295285 | 0.976 ( 0.885, 1.076 ) | 1.0000 |  | 2178/236252 | 1.022 ( 0.897, 1.164 ) | 1.0000 |
| LDL direct | 3812/322649 | 0.856 ( 0.826, 0.888 ) | **0.0000** |  | 2377/258369 | 0.915 ( 0.866, 0.967 ) | **0.0453** |
| Lipoprotein A | 3028/257032 | 1.000 ( 0.999, 1.001 ) | 1.0000 |  | 1904/206102 | 1.000 ( 0.999, 1.000 ) | 1.0000 |
| Triglycerides | 3794/320828 | 0.950 ( 0.916, 0.985 ) | 0.1826 |  | 2368/256997 | 0.936 ( 0.891, 0.983 ) | 0.2375 |
| Alanine aminotransferase | 3800/320834 | 0.992 ( 0.989, 0.996 ) | **0.0005** |  | 2369/256928 | 0.989 ( 0.985, 0.994 ) | **0.0002** |
| Albumin | 3488/295696 | 0.994 ( 0.981, 1.008 ) | 1.0000 |  | 2179/236605 | 0.995 ( 0.979, 1.012 ) | 1.0000 |
| Aspartate aminotransferase | 3773/320218 | 1.005 ( 1.001, 1.010 ) | 0.7529 |  | 2348/256514 | 1.002 ( 0.997, 1.008 ) | 1.0000 |
| Direct bilirubin | 3298/272548 | 1.018 ( 0.969, 1.069 ) | 1.0000 |  | 2091/219262 | 0.991 ( 0.931, 1.056 ) | 1.0000 |
| Gamma glutamyltransferase | 3770/320381 | 1.002 ( 1.001, 1.003 ) | **0.0108** |  | 2351/256718 | 1.001 ( 1.000, 1.003 ) | 1.0000 |
| Total bilirubin | 3788/318963 | 0.992 ( 0.983, 1.002 ) | 1.0000 |  | 2356/255285 | 0.995 ( 0.983, 1.006 ) | 1.0000 |
| Creatinine | 3788/322416 | 1.000 ( 0.997, 1.002 ) | 1.0000 |  | 2366/258291 | 0.998 ( 0.994, 1.001 ) | 1.0000 |
| Cystatin C | 3759/321904 | 1.727 ( 1.402, 2.127 ) | **0.0000** |  | 2352/258042 | 1.315 ( 0.987, 1.752 ) | 1.0000 |
| Phosphate | 3491/295269 | 1.066 ( 0.849, 1.338 ) | 1.0000 |  | 2180/236233 | 0.873 ( 0.654, 1.165 ) | 1.0000 |
| Total protein | 3494/295310 | 1.000 ( 0.992, 1.008 ) | 1.0000 |  | 2183/236317 | 0.994 ( 0.983, 1.004 ) | 1.0000 |
| Urate | 3815/322780 | 0.999 ( 0.999, 1.000 ) | **0.0031** |  | 2379/258495 | 0.999 ( 0.998, 0.999 ) | **0.0001** |
| Urea | 3782/322093 | 0.972 ( 0.948, 0.996 ) | 0.7138 |  | 2364/258077 | 0.985 ( 0.955, 1.017 ) | 1.0000 |

***^a^***Model 1 adjusted for demographic and temporal variables.

***^b^***Model 2 additionally adjusted for socioeconomic, lifestyle and medication variables.

P value column in bold indicates statistical significance at a Bonferroni adjusted p value < 0.05.

Abbreviations: CI, confidence interval; HR, hazard ratio;

## Supplemental Table 7. Association between serum laboratory test and incident Alzheimer's disease.

| Alzheimer's disease | Model 1*^a^* | | |  | Model 2*^b^* | | |
| --- | --- | --- | --- | --- | --- | --- | --- |
|  | Event No./Total No. | HR ( 95% CI ) | P value (Bonferroni) |  | Event No./Total No. | HR ( 95% CI ) | P value (Bonferroni) |
| Alkaline phosphatase | 2061/348548 | 0.998 ( 0.996, 1.000 ) | 1.0000 |  | 1263/279797 | 0.996 ( 0.993, 0.999 ) | 0.0800 |
| Calcium | 1876/319358 | 1.048 ( 0.639, 1.718 ) | 1.0000 |  | 1142/256079 | 0.711 ( 0.376, 1.345 ) | 1.0000 |
| Rheumatoid factor | 173/31158 | 1.003 ( 0.996, 1.011 ) | 1.0000 |  | 109/24666 | 1.009 ( 1.000, 1.018 ) | 1.0000 |
| Vitamin D | 1966/334008 | 0.996 ( 0.994, 0.998 ) | **0.0087** |  | 1209/268398 | 0.997 ( 0.994, 1.000 ) | 1.0000 |
| IGF-1 | 2061/347387 | 0.996 ( 0.988, 1.004 ) | 1.0000 |  | 1261/278765 | 1.002 ( 0.991, 1.012 ) | 1.0000 |
| Oestradiol | 138/53176 | 1.000 ( 0.999, 1.001 ) | 1.0000 |  | 79/47082 | 0.999 ( 0.998, 1.001 ) | 1.0000 |
| SHBG | 1847/315042 | 1.003 ( 1.002, 1.005 ) | **0.0155** |  | 1124/252489 | 1.002 ( 1.000, 1.005 ) | 1.0000 |
| Testosterone | 1788/315830 | 0.988 ( 0.971, 1.006 ) | 1.0000 |  | 1092/254489 | 0.992 ( 0.971, 1.015 ) | 1.0000 |
| Apolipoprotein A | 1865/318276 | 1.126 ( 0.940, 1.349 ) | 1.0000 |  | 1139/255138 | 1.026 ( 0.808, 1.303 ) | 1.0000 |
| Apolipoprotein B | 2058/348070 | 0.715 ( 0.598, 0.857 ) | **0.0080** |  | 1257/279297 | 0.900 ( 0.692, 1.172 ) | 1.0000 |
| Cholesterol | 2067/349606 | 0.937 ( 0.903, 0.972 ) | **0.0174** |  | 1263/280527 | 0.963 ( 0.910, 1.019 ) | 1.0000 |
| C-reactive protein | 2038/345075 | 1.002 ( 0.986, 1.018 ) | 1.0000 |  | 1248/277254 | 1.002 ( 0.980, 1.024 ) | 1.0000 |
| Glucose | 1840/316268 | 1.059 ( 1.004, 1.117 ) | 1.0000 |  | 1126/253883 | 1.041 ( 0.969, 1.118 ) | 1.0000 |
| Glycated haemoglobin | 2018/345985 | 1.015 ( 1.007, 1.024 ) | **0.0088** |  | 1234/277941 | 1.013 ( 1.001, 1.025 ) | 1.0000 |
| HDL cholesterol | 1878/319538 | 1.169 ( 1.027, 1.330 ) | 0.5389 |  | 1144/256183 | 1.044 ( 0.875, 1.246 ) | 1.0000 |
| LDL direct | 2065/348983 | 0.909 ( 0.866, 0.954 ) | **0.0032** |  | 1263/280026 | 0.950 ( 0.882, 1.024 ) | 1.0000 |
| Lipoprotein A | 1628/278061 | 1.000 ( 0.999, 1.001 ) | 1.0000 |  | 1005/223401 | 0.999 ( 0.998, 1.001 ) | 1.0000 |
| Triglycerides | 2056/347020 | 0.903 ( 0.857, 0.950 ) | **0.0027** |  | 1258/278539 | 0.905 ( 0.844, 0.971 ) | 0.1575 |
| Alanine aminotransferase | 2053/347011 | 0.989 ( 0.985, 0.994 ) | **0.0007** |  | 1257/278456 | 0.987 ( 0.981, 0.994 ) | **0.0059** |
| Albumin | 1875/319935 | 1.022 ( 1.003, 1.040 ) | 0.5796 |  | 1144/256538 | 1.020 ( 0.997, 1.044 ) | 1.0000 |
| Aspartate aminotransferase | 2041/346236 | 1.009 ( 1.003, 1.015 ) | 0.1229 |  | 1247/277925 | 1.003 ( 0.995, 1.011 ) | 1.0000 |
| Direct bilirubin | 1786/294794 | 1.079 ( 1.010, 1.153 ) | 0.7024 |  | 1111/237602 | 1.059 ( 0.974, 1.152 ) | 1.0000 |
| Gamma glutamyltransferase | 2047/346394 | 1.000 ( 0.999, 1.002 ) | 1.0000 |  | 1254/278150 | 0.999 ( 0.997, 1.001 ) | 1.0000 |
| Total bilirubin | 2053/344957 | 1.009 ( 0.997, 1.022 ) | 1.0000 |  | 1253/276665 | 1.011 ( 0.995, 1.026 ) | 1.0000 |
| Creatinine | 2051/348694 | 0.996 ( 0.993, 1.000 ) | 1.0000 |  | 1259/279932 | 0.994 ( 0.990, 0.999 ) | 0.5096 |
| Cystatin C | 2042/348070 | 1.029 ( 0.767, 1.379 ) | 1.0000 |  | 1255/279626 | 0.958 ( 0.637, 1.439 ) | 1.0000 |
| Phosphate | 1878/319520 | 0.919 ( 0.673, 1.253 ) | 1.0000 |  | 1145/256163 | 0.746 ( 0.499, 1.113 ) | 1.0000 |
| Total protein | 1880/319535 | 1.007 ( 0.996, 1.019 ) | 1.0000 |  | 1145/256229 | 1.002 ( 0.987, 1.016 ) | 1.0000 |
| Urate | 2063/349108 | 0.998 ( 0.998, 0.999 ) | **0.0000** |  | 1260/280162 | 0.998 ( 0.997, 0.999 ) | **0.0041** |
| Urea | 2048/348321 | 0.943 ( 0.911, 0.975 ) | **0.0209** |  | 1259/279690 | 0.976 ( 0.934, 1.019 ) | 1.0000 |

***^a^***Model 1 adjusted for demographic and temporal variables.

***^b^***Model 2 additionally adjusted for socioeconomic, lifestyle and medication variables.

P value column in bold indicates statistical significance at a Bonferroni adjusted p value < 0.05.

Abbreviations: CI, confidence interval; HR, hazard ratio;

## Supplemental Table 8. Association between serum laboratory test and incident vascular dementia.

| Vascular dementia | Model 1*^a^* | | |  | Model 2*^b^* | | |
| --- | --- | --- | --- | --- | --- | --- | --- |
|  | Event No./Total No. | HR ( 95% CI ) | P value (Bonferroni) |  | Event No./Total No. | HR ( 95% CI ) | P value (Bonferroni) |
| Alkaline phosphatase | 1020/347507 | 1.004 ( 1.002, 1.007 ) | **0.0330** |  | 593/279127 | 1.003 ( 0.999, 1.006 ) | 1.0000 |
| Calcium | 951/318433 | 1.384 ( 0.690, 2.777 ) | 1.0000 |  | 562/255499 | 0.679 ( 0.273, 1.690 ) | 1.0000 |
| Rheumatoid factor | 97/31082 | 0.998 ( 0.987, 1.010 ) | 1.0000 |  | 56/24613 | 0.996 ( 0.981, 1.013 ) | 1.0000 |
| Vitamin D | 976/333018 | 0.991 ( 0.988, 0.994 ) | **0.0000** |  | 563/267752 | 0.995 ( 0.991, 0.999 ) | 0.6142 |
| IGF-1 | 1028/346354 | 0.986 ( 0.974, 0.997 ) | 0.4904 |  | 597/278101 | 0.997 ( 0.982, 1.012 ) | 1.0000 |
| Oestradiol | 70/53108 | 1.000 ( 0.998, 1.001 ) | 1.0000 |  | 41/47044 | 1.000 ( 0.997, 1.002 ) | 1.0000 |
| SHBG | 941/314136 | 1.002 ( 0.999, 1.005 ) | 1.0000 |  | 557/251922 | 1.007 ( 1.003, 1.011 ) | **0.0157** |
| Testosterone | 928/314970 | 0.966 ( 0.944, 0.988 ) | 0.0800 |  | 540/253937 | 0.998 ( 0.969, 1.028 ) | 1.0000 |
| Apolipoprotein A | 948/317359 | 0.549 ( 0.419, 0.720 ) | **0.0004** |  | 557/254556 | 0.947 ( 0.668, 1.341 ) | 1.0000 |
| Apolipoprotein B | 1027/347039 | 0.368 ( 0.282, 0.479 ) | **0.0000** |  | 596/278636 | 0.800 ( 0.540, 1.185 ) | 1.0000 |
| Cholesterol | 1030/348569 | 0.795 ( 0.753, 0.839 ) | **0.0000** |  | 599/279863 | 0.979 ( 0.900, 1.064 ) | 1.0000 |
| C-reactive protein | 1020/344057 | 1.041 ( 1.022, 1.061 ) | **0.0008** |  | 594/276600 | 1.052 ( 1.027, 1.078 ) | **0.0014** |
| Glucose | 907/315335 | 1.256 ( 1.178, 1.339 ) | **0.0000** |  | 534/253291 | 1.124 ( 1.029, 1.227 ) | 0.2861 |
| Glycated haemoglobin | 1005/344972 | 1.063 ( 1.054, 1.073 ) | **0.0000** |  | 585/277292 | 1.039 ( 1.024, 1.055 ) | **0.0000** |
| HDL cholesterol | 949/318609 | 0.704 ( 0.578, 0.857 ) | **0.0142** |  | 556/255595 | 1.171 ( 0.905, 1.514 ) | 1.0000 |
| LDL direct | 1027/347945 | 0.737 ( 0.686, 0.791 ) | **0.0000** |  | 597/279360 | 0.946 ( 0.846, 1.058 ) | 1.0000 |
| Lipoprotein A | 822/277255 | 1.000 ( 0.998, 1.001 ) | 1.0000 |  | 480/222876 | 1.000 ( 0.998, 1.002 ) | 1.0000 |
| Triglycerides | 1024/345988 | 1.024 ( 0.958, 1.095 ) | 1.0000 |  | 594/277875 | 0.927 ( 0.842, 1.021 ) | 1.0000 |
| Alanine aminotransferase | 1025/345983 | 0.999 ( 0.993, 1.005 ) | 1.0000 |  | 597/277796 | 0.991 ( 0.982, 1.000 ) | 1.0000 |
| Albumin | 951/319011 | 0.967 ( 0.943, 0.992 ) | 0.2750 |  | 560/255954 | 0.976 ( 0.944, 1.008 ) | 1.0000 |
| Aspartate aminotransferase | 1015/345210 | 1.009 ( 1.000, 1.017 ) | 1.0000 |  | 591/277269 | 1.010 ( 1.000, 1.021 ) | 1.0000 |
| Direct bilirubin | 907/293915 | 1.006 ( 0.916, 1.105 ) | 1.0000 |  | 538/237029 | 0.940 ( 0.830, 1.066 ) | 1.0000 |
| Gamma glutamyltransferase | 1014/345361 | 1.005 ( 1.003, 1.007 ) | **0.0000** |  | 589/277485 | 1.003 ( 1.001, 1.006 ) | 0.3835 |
| Total bilirubin | 1023/343927 | 0.982 ( 0.964, 1.000 ) | 1.0000 |  | 595/276007 | 0.987 ( 0.964, 1.011 ) | 1.0000 |
| Creatinine | 1011/347654 | 1.001 ( 0.996, 1.005 ) | 1.0000 |  | 590/279263 | 0.996 ( 0.990, 1.003 ) | 1.0000 |
| Cystatin C | 1003/347031 | 3.297 ( 2.246, 4.842 ) | **0.0000** |  | 586/278957 | 1.904 ( 1.112, 3.262 ) | 0.5688 |
| Phosphate | 952/318594 | 0.992 ( 0.642, 1.533 ) | 1.0000 |  | 561/255579 | 0.817 ( 0.464, 1.437 ) | 1.0000 |
| Total protein | 951/318606 | 1.003 ( 0.987, 1.019 ) | 1.0000 |  | 560/255644 | 0.992 ( 0.971, 1.012 ) | 1.0000 |
| Urate | 1025/348070 | 1.000 ( 0.999, 1.001 ) | 1.0000 |  | 598/279500 | 0.999 ( 0.998, 1.000 ) | 1.0000 |
| Urea | 1011/347284 | 0.988 ( 0.943, 1.036 ) | 1.0000 |  | 590/279021 | 0.982 ( 0.924, 1.045 ) | 1.0000 |

***^a^***Model 1 adjusted for demographic and temporal variables.

***^b^***Model 2 additionally adjusted for socioeconomic, lifestyle and medication variables.

P value column in bold indicates statistical significance at a Bonferroni adjusted p value < 0.05.

Abbreviations: CI, confidence interval; HR, hazard ratio;

## Supplemental Table 9. Linear and nonlinear associations between serum laboratory test and risk of incident dementia after excluding participants with comorbidities.

|  | Model 2*^a^* | | | | |
| --- | --- | --- | --- | --- | --- |
|  | Event No./Total No. | HR ( 95% CI ) | P value (Bonferroni) |  | P non-linear (Bonferroni) |
| **Bone and joint *^b^*** |  |  |  |  |  |
| Alkaline phosphatase | 2674/272527 | 0.999 ( 0.998, 1.001 ) | 1.0000 |  | 1.0000 |
| Calcium | 2456/249464 | 0.548 ( 0.353, 0.850 ) | 0.2185 |  | 1.0000 |
| Rheumatoid factor | 261/23986 | 1.003 ( 0.996, 1.009 ) | 1.0000 |  | 1.0000 |
| Vitamin D | 2562/261493 | 0.994 ( 0.992, 0.996 ) | **0.0000** |  | 0.1359 |
| **Endocrine *^c^*** |  |  |  |  |  |
| IGF-1 | 2780/277979 | 1.007 ( 0.999, 1.014 ) | 1.0000 |  | **0.0000** |
| Oestradiol | 203/46492 | 1.000 ( 0.999, 1.000 ) | 1.0000 |  | 1.0000 |
| SHBG | 2523/251825 | 1.004 ( 1.003, 1.006 ) | **0.0001** |  | 1.0000 |
| Testosterone | 2479/253954 | 1.000 ( 0.986, 1.015 ) | 1.0000 |  | **0.0072** |
| **Immunometabolic *^d^*** |  |  |  |  |  |
| Apolipoprotein A | 1885/223535 | 0.992 ( 0.824, 1.196 ) | 1.0000 |  | 1.0000 |
| Apolipoprotein B | 2064/244850 | 0.771 ( 0.628, 0.945 ) | 0.3742 |  | 1.0000 |
| Cholesterol | 2070/245862 | 0.950 ( 0.909, 0.993 ) | 0.6737 |  | 1.0000 |
| C-reactive protein | 2042/243079 | 1.018 ( 1.001, 1.034 ) | 1.0000 |  | **0.0000** |
| Glucose | 1891/224056 | 1.093 ( 1.027, 1.163 ) | 0.1548 |  | 1.0000 |
| Glycated haemoglobin | 2069/245216 | 1.006 ( 0.995, 1.017 ) | 1.0000 |  | 1.0000 |
| HDL cholesterol | 1888/224475 | 1.040 ( 0.906, 1.194 ) | 1.0000 |  | 1.0000 |
| LDL direct | 2069/245435 | 0.932 ( 0.880, 0.987 ) | 0.4899 |  | 1.0000 |
| Lipoprotein A | 1675/197131 | 0.999 ( 0.998, 1.000 ) | 1.0000 |  | 1.0000 |
| Triglycerides | 2067/244355 | 0.917 ( 0.868, 0.969 ) | 0.0632 |  | 1.0000 |
| **Liver *^e^*** |  |  |  |  |  |
| Alanine aminotransferase | 2773/278431 | 0.990 ( 0.986, 0.995 ) | **0.0003** |  | **0.0082** |
| Albumin | 2554/256514 | 0.997 ( 0.982, 1.013 ) | 1.0000 |  | 0.2167 |
| Aspartate aminotransferase | 2749/277910 | 1.004 ( 0.999, 1.009 ) | 1.0000 |  | 1.0000 |
| Direct bilirubin | 2446/237592 | 0.998 ( 0.942, 1.058 ) | 1.0000 |  | 1.0000 |
| Gamma glutamyltransferase | 2749/278167 | 1.002 ( 1.000, 1.003 ) | 0.4063 |  | 1.0000 |
| Total bilirubin | 2758/276622 | 0.994 ( 0.984, 1.005 ) | 1.0000 |  | 1.0000 |
| **Renal *^f^*** |  |  |  |  |  |
| Creatinine | 2750/278860 | 0.997 ( 0.994, 1.000 ) | 1.0000 |  | **0.0066** |
| Cystatin C | 2733/278613 | 1.440 ( 1.101, 1.884 ) | 0.2342 |  | **0.0199** |
| Phosphate | 2534/255024 | 0.840 ( 0.642, 1.099 ) | 1.0000 |  | 1.0000 |
| Total protein | 2535/255080 | 0.998 ( 0.988, 1.008 ) | 1.0000 |  | 1.0000 |
| Urate | 2756/278877 | 0.998 ( 0.998, 0.999 ) | **0.0000** |  | **0.0269** |
| Urea | 2744/278623 | 0.983 ( 0.954, 1.012 ) | 1.0000 |  | **0.0000** |

***^a^***Model 2 adjusted for demographic, temporal, socioeconomic, lifestyle and medication variables.

***^b^*** Individuals with disorders of bone density and structure (ICD-10 codes of M80-M85), other osteopathies (ICD-10 codes of M86-M90) and chondropathies (ICD-10 codes of M91-M94) were excluded for markers in the “bone and joint” category.

***^c^*** Individuals with disorders of thyroid gland (ICD-10 codes of E00-E07), and disorders of other endocrine glands (ICD-10 codes of E20-E35) were excluded for markers in the “endocrine” category.

***^d^*** Individuals with certain disorders involving the immune mechanism (ICD-10 codes of D80-D89), diabetes mellitus (ICD-10 codes of E10-E14), other disorders of glucose regulation and pancreatic internal secretion (ICD-10 codes of E15-E16), obesity and other hyperalimentation (ICD-10 codes of E65-E68), metabolic disorders (ICD-10 codes of E70-E90) were excluded for markers in the “immunometabolic” category.

***^e^*** Individuals with glomerular diseases (ICD-10 codes of K70-K77), renal tubulo-interstitial diseases, renal failure were excluded for markers in the “liver” category.

***^f^*** Individuals with glomerular diseases (ICD-10 codes of N00-N08), renal tubulo-interstitial diseases (ICD-10 codes of N10-N16), renal failure (ICD-10 codes of N17-N19) were excluded for markers in the “renal” category.

P value column in bold indicates statistical significance at a Bonferroni adjusted p value < 0.05.

Abbreviations: CI, confidence interval; HR, hazard ratio;

## Supplemental Figure1. Pearson correlation coefficients for each pair of serum laboratory tests.


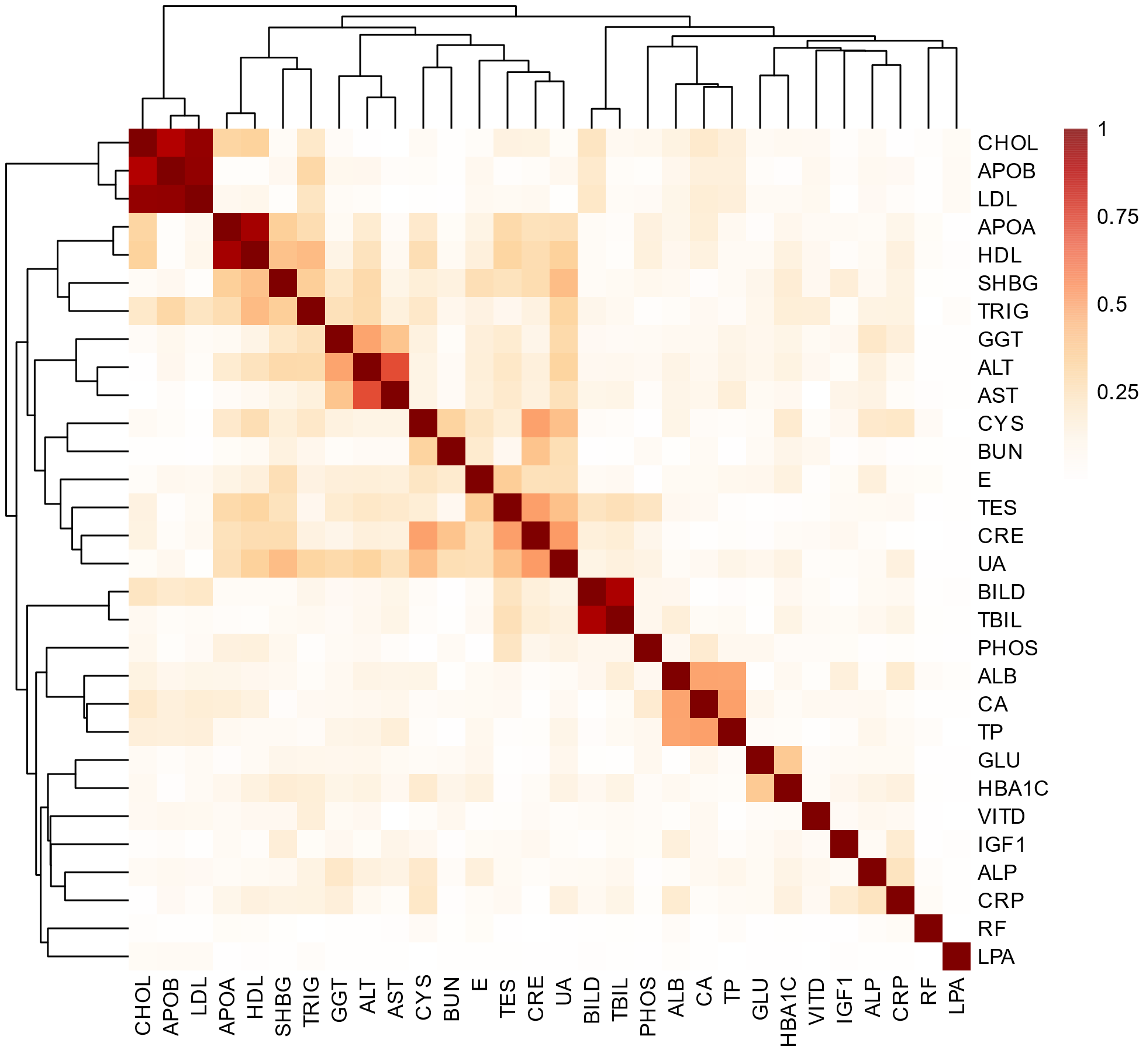


For each pair of tests, the correlation is taken across all Europe participants without dementia at baseline.

Abbreviations: ALP, Alkaline phosphatase; CA, Calcium; RF, Rheumatoid factor; VITD, Vitamin D; IGF1, IGF-1; E, Oestradiol; SHBG, Sex hormone-binding globulin; TES, Testosterone; APOA, Apolipoprotein A; APOB, Apolipoprotein B; CHOL, Cholesterol; CRP, C-reactive protein; GLU, Glucose; HBA1C, Glycated haemoglobin; HDL, HDL cholesterol; LDL, LDL cholesterol; LPA, Lipoprotein A; TRIG, Triglycerides; ALT, Alanine aminotransferase; ALB, Albumin; AST, Aspartate aminotransferase; BILD, Direct bilirubin; GGT, Gamma glutamyltransferase; TBIL, Total bilirubin; CRE, Creatinine; CYS, Cystatin C; PHOS, Phosphate; TP, Total protein; UA, Urate; BUN, Urea;

## Supplemental Figure2. Continuation of Figure 2.


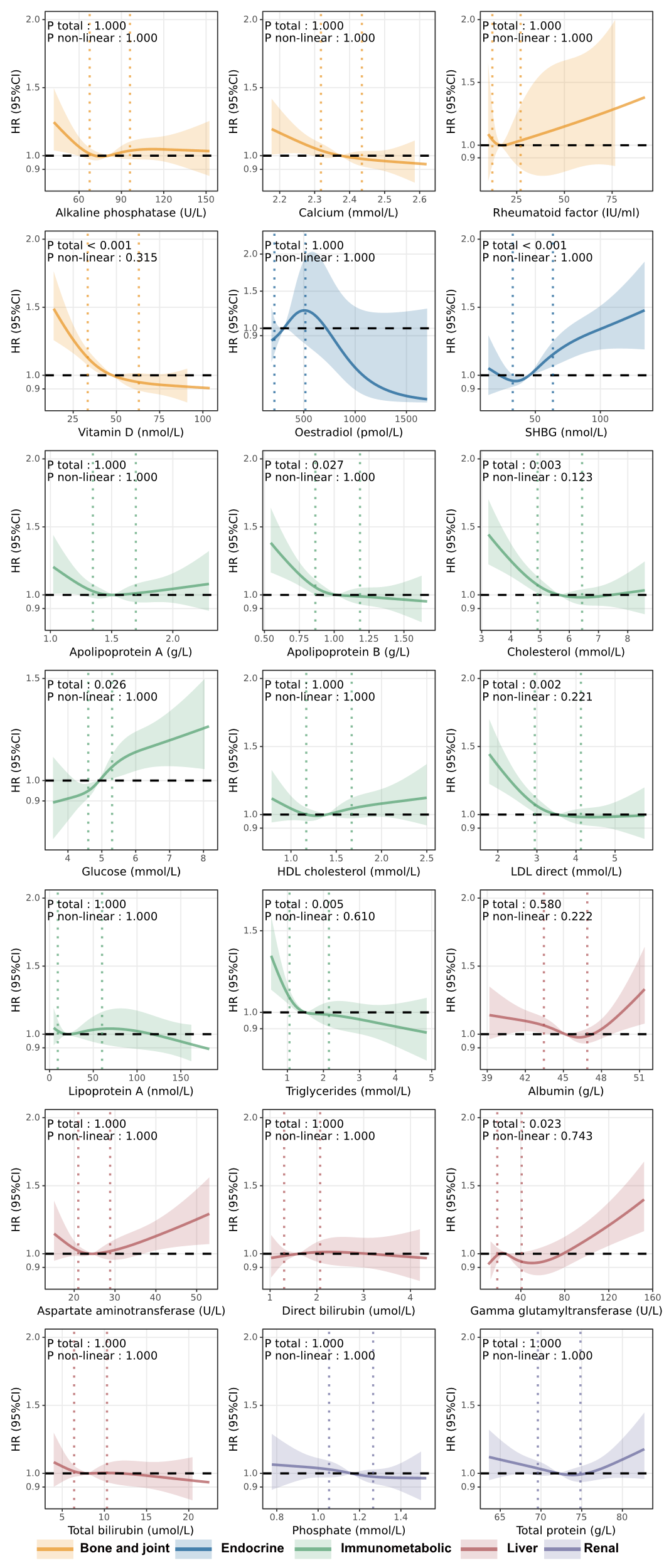


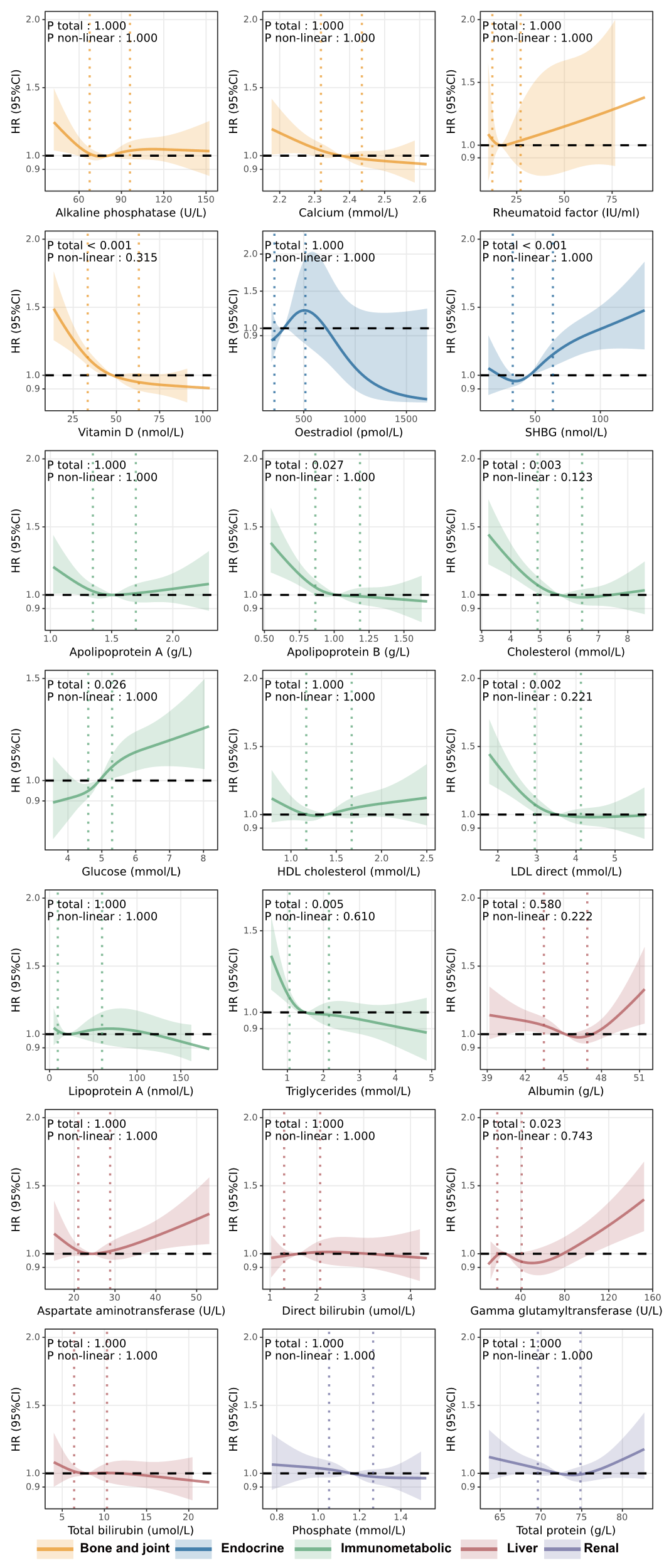


The yellow, blue, green, red and purple indicates each of serum laboratory tests fitting into “Bone and joint”, “endocrine”, “immunometabolic”, “liver” and “renal” category.
